# Ferroptosis MRI for early detection of anticancer drug–induced acute cardiac/kidney injuries

**DOI:** 10.1126/sciadv.add8539

**Published:** 2023-03-08

**Authors:** Fantian Zeng, Sureya Nijiati, Yangtengyu Liu, Qinqin Yang, Xiaomin Liu, Qianyu Zhang, Shi Chen, Anqi Su, Hehe Xiong, Changrong Shi, Congbo Cai, Zhongning Lin, Xiaoyuan Chen, Zijian Zhou

**Affiliations:** ^1^ State Key Laboratory of Molecular Vaccinology and Molecular Diagnostics & Center for Molecular Imaging and Translational Medicine, School of Public Health, Xiamen University, Xiamen 361102, China.; ^2^ Department of Rheumatology and Immunology, Xiangya Hospital, Central South University, Changsha 410008, China.; ^3^ Department of Electronic Science, Fujian Provincial Key Laboratory of Plasma and Magnetic Resonance, Xiamen University, Xiamen 361102, China.; ^4^ Departments of Diagnostic Radiology, Chemical and Biomolecular Engineering, and Biomedical Engineering, Yong Loo Lin School of Medicine and College of Design and Engineering, National University of Singapore, Singapore 119074, Singapore.; ^5^ Clinical Imaging Research Centre, Centre for Translational Medicine, Yong Loo Lin School of Medicine, National University of Singapore, Singapore 117599, Singapore.; ^6^ Nanomedicine Translational Research Programme, NUS Center for Nanomedicine, Yong Loo Lin School of Medicine, National University of Singapore, Singapore 117597, Singapore.; ^7^ Shenzhen Research Institute of Xiamen University, Shenzhen 518057, China.

## Abstract

Ferroptosis has been realized in anticancer drug–induced acute cardiac/kidney injuries (ACI/AKI); however, molecular imaging approach to detect ferroptosis in ACI/AKI is a challenge. We report an artemisinin-based probe (Art-Gd) for contrast-enhanced magnetic resonance imaging of ferroptosis (feMRI) by exploiting the redox-active Fe(II) as a vivid chemical target. In vivo, the Art-Gd probe showed great feasibility in early diagnosis of anticancer drug–induced ACI/AKI, which was at least 24 and 48 hours earlier than the standard clinical assays for assessing ACI and AKI, respectively. Furthermore, the feMRI was able to provide imaging evidence for the different mechanisms of action of ferroptosis-targeted agents, either by blocking lipid peroxidation or depleting iron ions. This study presents a feMRI strategy with simple chemistry and robust efficacy for early evaluation of anticancer drug–induced ACI/AKI, which may shed light on the theranostics of a variety of ferroptosis-related diseases.

## Teaser

Molecular imaging of ferroptosis by exploiting Fe(II) as a vivid chemical target is achieved by an Artemisinin-based MRI probe.

## INTRODUCTION

Chemotherapy is still the predominant first-line cancer treatment option in the clinic (*1*). However, one of the major caveats to chemotherapy is the side toxicity from the systemic administration of therapeutic drugs, which often leads to poor prognosis with severe clinical complications in patients (*2*, *3*). For example, acute cardiac injury (ACI) and acute kidney injury (AKI) are two common cases in patients due to the specialized roles of the heart and kidneys in circulation and excretion, respectively (*4*, *5*). Specifically, the high incidence and mortality rates of both ACI and AKI necessitate the need for early detection of the dysfunction of these organs during cancer treatment. Mounting evidence has shown that early intervention, especially at the incipient stage of ACI/AKI, could largely prevent the progression and reduce the mortality (*6*). Current diagnoses of ACI and AKI in the clinic are based on the detection of cardiac troponin (cTn) (*7*) and serum creatinine (sCr) (*8*), respectively. However, these two biomarkers are mostly detectable at a relatively late stage of ACI/AKI in which the tissue damages have already occurred. As a result, the delayed diagnosis of ACI/AKI poses a great challenge to reversing the pathological processes in cardiac and kidney tissues. Recently, several biomarkers, such as creatine kinase isoenzymes (CK-MB) and lactate dehydrogenase (LDH) for ACI (*9*) and neutrophil gelatinase–associated lipocalin (NGAL) and kidney injury molecule-1 (KIM-1) for AKI (*10*, *11*), have shown promises in the early diagnosis of ACI/AKI. However, these diagnostic methods involve invasive samplings and lack tissue level specificity to define the distribution and status of the lesions.

Ferroptosis is a regulated form of cell death characterized by iron-dependent lipid peroxidation on cell membranes (*12*, *13*). Recently, increasing evidences have revealed that ferroptosis is closely related to the pathological progression of anticancer drug–induced ACI and AKI (*14*–*16*). Some features distinctly related to ferroptosis, such as abnormal iron metabolism and lipid peroxidation, were identified in doxorubicin (DOX)–induced cardiomyopathy (*17*, *18*) and cisplatin (CDDP)–induced nephropathy (*19*, *20*). Further studies showed that ferroptosis inhibitors could obviously alleviate the anticancer drug–induced ACI and AKI (*21*, *22*). Therefore, the diagnostic imaging and therapeutic planning of anticancer drug–induced cardiomyopathy and nephropathy could benefit from realizing ferroptosis as a mechanism of target. To this end, we hypothesized that redox-active Fe(II), the key modulator in ferroptosis, may serve as a vivid chemical target for ferroptosis-related imaging and treatment.

Magnetic resonance imaging (MRI) is of great importance in depicting the anatomical structures of soft tissues in a noninvasive and comprehensive manner. The signal of MRI mostly comes from water protons in the body, which often requires contrast-enhancement mechanisms (e.g., contrast agents and susceptibility mapping) to improve the specificity in clinical diagnosis. For example, late gadolinium enhancement (LGE) MRI is a widely accepted means of assessing the structural changes of cardiac and kidney tissues in the clinic (*23*, *24*). However, LGE MRI has limited value in the ACI/AKI diagnosis due to the inability to detect early changes at molecular level. Quantitative susceptibility mapping (QSM) by MRI is an emerging technique for studying the spatial distribution of magnetic susceptibility of dominant iron sources (i.e., ferritins) in living body (*25*). The most readily translatable applications of QSM are, among others, the separation of diamagnetic calcium from paramagnetic irons and the quantification of iron deposition in tissues. However, it is still challenging to distinguish between Fe(II) and Fe(III) using QSM regardless of the convoluted data acquisition and processing (*26*), giving prominence to develop molecular imaging probes enabling to map the Fe(II) in living individuals.

**Fig. 1.**
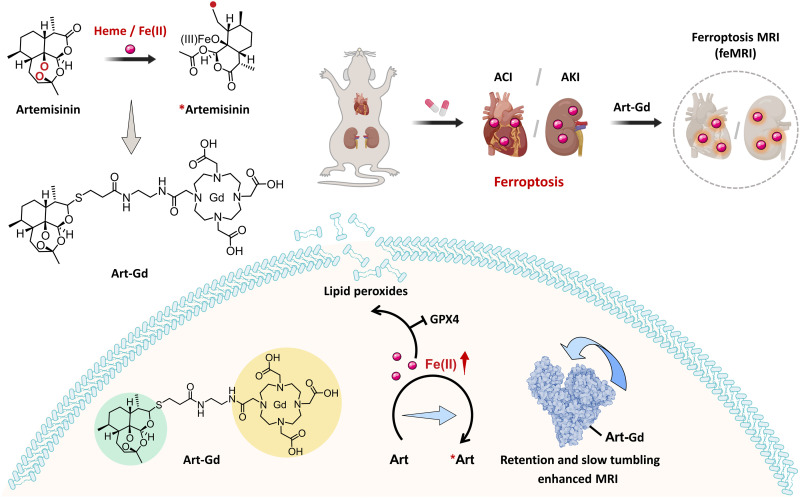
Illustration of the Art-Gd probe for feMRI of anticancer drug–induced ACI/AKI. Learning from the Fe(II)-specific activation mechanism of artemisinin, the Art-Gd probe is designed to magnetic resonance imaging of ferroptosis (feMRI) targeting the reactivity of Fe(II). Ferroptosis is involved in the pathogenesis of doxorubicin (DOX)–induced acute cardiac injury (ACI) and cisplatin (CDDP)–induced acute kidney injury (AKI). The Fe(II) in the cardiac and renal cells could serve as a chemical target for the early evaluation of anticancer drug–induced ACI/AKI. The radical production of the Art-Gd probe in the presence of Fe(II) leads to the formation of Art-Gd protein complexes, resulting in the enhanced *T*_1_ MRI contrast efficiency due to the retention effect and the slow tumbling effect. GPX 4, glutathione peroxidase 4.

Inspired by the antimalarial mechanism of artemisinin that the endoperoxide bridge structure reacts with the metabolic products of Fe(II)-porphyrin to afford radicals (*27*), we here designed an artemisinin-based MRI probe by chelating gadolinium ions [Gd(III)] through 1,4,7,10-tetraazacyclododecane–1,4,7,10-tetraacetic acid (DOTA) group, denoted as Art-Gd (Fig. 1). Previous efforts have been focused on the fabrication of a 1,2,4-trioxolane (TRX) moiety, mimicking the endoperoxide bridge structure of artemisinin to afford Fe(II)-promoted fragmentation property (*28*). A caging group is often involved with a multistep synthetic chemistry to develop fluorescence (*28*) and positron emission tomography (PET) imaging probes (*29*, *30*). Here, we turned to directly conjugating Gd species to artemisinin through a simple chemistry, taking advantages of the reactivity-based self-enhancement mechanism of the Fe(II)-responsive MRI of ferroptosis, denoted as feMRI hereafter. In the presence of Fe(II), the Art-Gd molecules afford the carbon-centered radicals, which are prone to attach to nearby proteins or lipid membranes. This phenomenon leads to two distinct features of the Art-Gd molecules: (i) the enhanced retention effect in the tissues and (ii) the enhanced longitudinal relaxation time (*T*_1_) contrast due to the slow tumbling effect of the entity. We studied the radical formation of the Art-Gd probe and confirmed the *T*_1_ MRI contrast enhancement in ferroptosis both in vitro and in vivo. After confirming the DOX-induced ACI and CDDP-induced AKI in mouse models, we studied the feasibility of using feMRI for early detection of the ACI and AKI. The feMRI showed that ferrostatin-1 (Fer-1) and desferrioxamine (DFO), two commonly used ferroptosis-targeting agents capable of blocking lipid peroxidation and depleting iron ions, respectively, had distinctly different protection efficiencies against the CDDP-induced AKI in mouse model. This work presents a simple but robust Fe(II)-targeted feMRI probe for the early evaluation of anticancer drug–induced ACI and AKI.

## RESULTS

### Characterizations of the Art-Gd probe

**Fig. 2.**
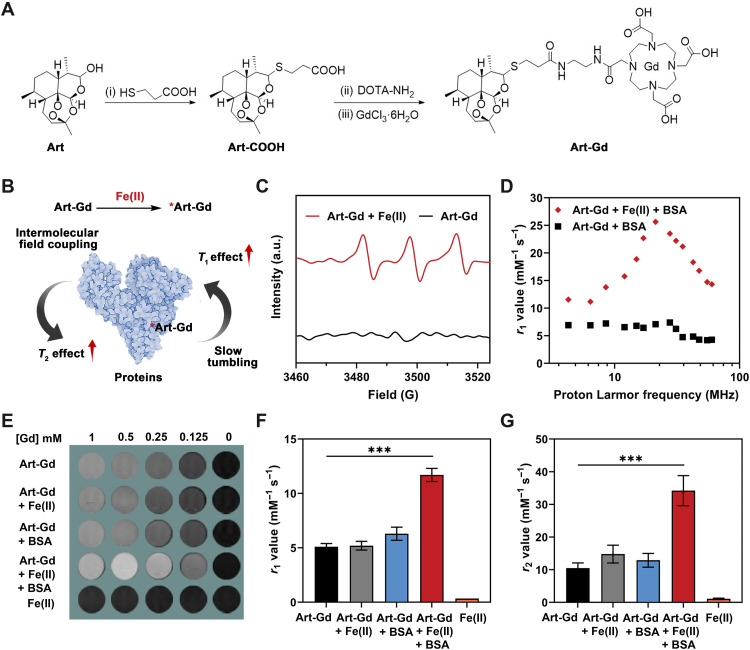
Characterizations of the Art-Gd probe. (**A**) Chemical structures and synthetic routes of the Art-Gd probe. DOTA-NH_2_, 2-aminoethyl-monoamide–1,4,7,10-tetraazacyclododecane–1,4,7,10-tetraacetic acid. (**B**) Schematic illustration of the mechanism of MRI enhancement of the Art-Gd probe. (**C**) Electron paramagnetic resonance (EPR) spectra of the Art-Gd probe with or without Fe(II) (1 mM FeCl_2_). a.u., arbitrary units. (**D**) The *T*_1_ nuclear magnetic resonance dispersion (NMRD) profiles of the Art-Gd + bovine serum albumin (BSA) in the presence (red) and absence (black) of Fe(II). (**E**) Representative *T*_1_ MRI phantoms of Art-Gd, Art-Gd + Fe(II), Art-Gd + BSA, Art-Gd + Fe(II) + BSA, and FeCl_2_ at different concentrations (1, 0.5, 0.25, 0.125, and 0 mM, respectively). (**F** and **G**) Columns show the *T*_1_ relaxivity (*r*_1_) and *T*_2_ relaxivity (*r*_2_) values of Art-Gd, Art-Gd + Fe(II), Art-Gd + BSA, Art-Gd + Fe(II) + BSA, and FeCl_2_. ***P < 0.001.

The Art-Gd molecules were obtained by an optimized three-step synthetic route starting with dihydroartemisinin (DHA) (Fig. 2A): (i) DHA was coupled with 3-mercaptocarboxylic acid to afford Art-S-COOH; (ii) condensation reaction with 2-aminoethyl-monoamide-DOTA (DOTA-NH_2_) afforded Art-S-DOTA; and (iii) chelating paramagnetic Gd(III) ions yielded the Art-Gd molecules. The products for each step were confirmed by ^1^H nuclear magnetic resonance (NMR), ^13^C NMR, and mass spectrometry (MS) (figs. S1 to S6). The physiological stability of the Art-S-DOTA molecules was tested and compared with the Art-O-DOTA molecules, which contain an ester bond between artemisinin and DOTA groups (figs. S7 to S9). The two compounds were labeled with gallium-68 (^68^Ga), incubated with mouse plasma at 37°C and analyzed by radio high-performance liquid chromatography (radio-HPLC). The results showed that the ^68^Ga-labeled Art-O-DOTA (denoted as Art-O-^68^Ga) molecules were easily degraded to the purity of 22.1 ± 3.1 and 10.2 ± 1.2% after 30 and 60 min of incubation, respectively (fig. S10). In contrast, the ^68^Ga-labeled Art-S-DOTA (denoted as Art-S-^68^Ga) molecules had negligible degradation with the purity of 97.2 ± 2.2% after 60 min of incubation.

To reveal the reactivity of the Art-Gd probe with Fe(II), we performed MRI phantom study on a 9.4-T scanner. We anticipated that the Art-Gd molecules could form the radicals in the presence of Fe(II) and subsequently attach onto biological substances (e.g., proteins or lipid membranes) through the formation of covalent bonds in a nonspecific manner (Fig. 2B). As a result, the slow tumbling feature of the obtained complexes would show increased molecular rotation time (τ_R_) and enhanced *T*_1_ relaxivity according to the chemical exchange mechanism (*31*, *32*). Meanwhile, the intermolecular field coupling of the magnetic centers [Gd(III)] upon complexation with the biological substances may cause augmented local field inhomogeneity, which may lead to slightly enhanced transverse relaxation time (*T*_2_) relaxivity (*33*, *34*). To test this hypothesis, we first used 5,5-dimethyl-1-pyrroline N-oxide (DMPO) as a spin trapping agent to identify the formation of radicals for the Art-Gd molecules in the presence of Fe(II) by electron paramagnetic resonance (EPR) measurements. The prominent EPR signal was observed for the Art-Gd + Fe(II), indicating the formation of the *Art-Gd radicals (Fig. 2C). Next, the *T*_1_ nuclear magnetic resonance dispersion (NMRD) profile of the Art-Gd molecules in the presence of both Fe(II) and bovine serum albumin (BSA) showed a “hump” with the proton Larmor frequency at around 21 MHz (Fig. 2D). This phenomenon is a typical feature of slow tumbling macromolecules in their relaxation enhancement, which further implies the formation of Art-Gd–BSA complexes. The *T*_1_ and *T*_2_ phantoms of different groups at different Gd concentrations (1, 0.5, 0.25, 0.125, and 0 mM) were shown in the Fig. 2E and fig. S11. The Art-Gd probe in the presence of Fe(II) and BSA showed a 2.1-fold enhancement of the *r*_1_ relaxivity value (10.70 ± 0.60 mM^−1^ s^−1^) compared with that of the Art-Gd molecules alone (5.10 ± 0.30 mM^−1^ s^−1^), while the variation of *r*_1_ relaxivity values between the Art-Gd + Fe(II) (5.20 ± 0.40 mM^−1^ s^−1^) and the Art-Gd + BSA (6.30 ± 0.60 mM^−1^ s^−1^) was not significant (Fig. 2F). These results are consistent with the contrast performance in the *T*_1_ phantom images. Similarly, the Art-Gd + Fe(II) + BSA had an increased *r*_2_ relaxivity value of 33.20 ± 4.60 mM^−1^ s^−1^, which was 2.3-fold higher than that of the Art-Gd molecules alone (10.50 ± 1.60 mM^−1^ s^−1^), ascribing to the intermolecular field coupling effect (Fig. 2G). Furthermore, the selectivity of the Art-Gd probe to Fe(II) was confirmed by measuring the *r*_1_ relaxivity value changes with other biologically relevant Cu(II), Ca(II), Mg(II), Fe(III), Zn(II), and heme. The results showed that heme had a similar effect with Fe(II) on the *r*_1_ relaxivity value changes, while the other ions had little to no effect (figs. S12 and S13). In addition, the *r*_1_ and *r*_2_ relaxivity values of the gadopentetic acid (Magnevist, Gd-DTPA) were not changed in the presence of BSA and Fe(II)/heme (fig. S14). Note that the low *r*_2_/*r*_1_ ratios (less than 3) of the Art-Gd probe in various formulas indicate the favorable contrast enhancement tendency in *T*_1_-weighted MRI (table S1). Because the endogenous Fe(II) with redox activity also have the ability to promote oxidative stress by participating in processes like the Fenton reaction, we further investigated the reactivity of the DHA to Fe(II) under the Fenton reaction conditions by MS analysis using α-(4-pyridyl-1-oxide)-*N*-tert-butylnitrone (4-POBN) as a carbon radical trapping agent. The mass/charge ratio (*m*/*z*) peak of 403 of the 4-POBN/DHA radical adduct was maintained in the presence of H_2_O_2_ (100 μM) (fig. S15), indicating that the reaction between the Fe(II) of the Fenton reaction and the carbon radical formation of the DHA structure was not influenced. Together, the Art-Gd probe is capable of responding to Fe(II) with high selectivity via the reactivity-based self-enhancement mechanism in MRI.

To assess the cytotoxicity of the Art-Gd probe, murine cardiac H9c2 and human embryonic kidney (HEK) 293T cells were incubated with different concentrations of the Art-Gd probe for 48 hours. The cell viability results showed that 82.8 ± 1.9% of H9c2 cells and 91.4 ± 2.8% of HEK293T cells were alive with the concentration of the Art-Gd up to 200 μM (fig. S16), indicating the good biocompatibility of the Art-Gd probe. Next, to investigate the feasibility of the feMRI of the Art-Gd probe in vitro, we preincubated H9c2 and HEK293T cells with erastin (a ferroptosis inducer) and used an Fe(II)-sensitive fluorescence imaging probe (i.e., FerroOrange) to determine the intracellular concentration of Fe(II) by flow cytometry. The results showed that the maximal fluorescence intensity was attained at 8 and 12 hours after incubation with H9c2 and HEK293T cells, respectively (fig. S17). To further determine the optimal time window for probing the Fe(II) in vitro, we evaluated the uptake of the Art-Gd probe in erastin-treated H9c2 and HEK293T cells at the respective 8 and 12 hours post-incubation time points by the inductively coupled plasma atomic emission spectrometry. The intracellular uptake of Gd(III) peaked at 1 hour for both cells pretreated with erastin (fig. S18). Subsequently, we conducted MRI to track the changes of *T*_1_ relaxation time in ferroptotic cells using the Art-Gd probe. The *T*_1_ map results showed that the erastin + Art-Gd groups received a prominent *T*_1_ relaxation time change, which was significantly higher than that of the Art-Gd probe alone in both H9c2 (1313.0 ± 41.9 versus 996.5 ± 20.0 ms) and HEK293T cells (898.4 ± 84.7 versus 597.8 ± 33.7 ms), respectively (**P = 0.0047 and ***P = 0.0003; figs. S19 and S20). Moreover, the *T*_1_ relaxation time changes of the erastin + Art-Gd group could be restored by Fer-1 treatment. Collectively, these results demonstrate that the Fe(II)-targeted Art-Gd probe holds great promise for feMRI in vitro, which is amenable to the noninvasive detection of ferroptosis-related diseases in vivo.

### The feMRI in DOX-induced mouse ACI model

**Fig. 3.**
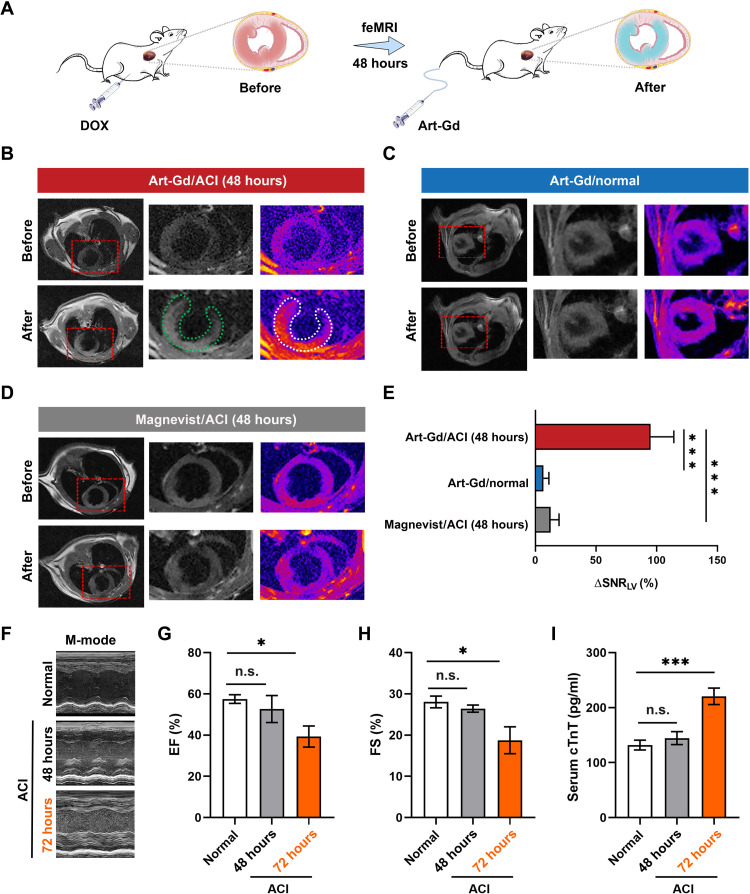
The feMRI in DOX-induced ACI mouse model. (**A**) Scheme shows that mice were administered with doxorubicin (DOX) (10 mg/kg) by intraperitoneal injection. After 48 hours, the feMRI was performed by intravenous injection of the Art-Gd probe. (**B** to **D**) Representative *T*_1_-weighted images at pre- and postcontrast time points for different groups (*n* = 3 per group), including Art-Gd/ACI, Art-Gd/normal, and Magnevist/ACI. The MR images were acquired at the same in-plane axial slices through a prefixed catheter. Red dashed rectangles indicate the axial area of the heart. Green and white dashed circles highlight the contrast areas for the black and white image and the corresponding pseudo-color image, respectively. (**E**) Quantification of ΔSNR (Δsignal-to-noise ratio) of the left ventricular (LV) from the *T*_1_-weighted images (***P < 0.001). (**F**) Representative echocardiograms from the DOX-induced ACI and normal mice at 48 and 72 hours. M-mode, motion mode. (**G** and **H**) The LV ejection fraction (EF) and the LV fractional shortening (FS) of heart from the DOX-induced ACI (48 and 72 hours) and normal mice (*n* = 3). (**I**) The serum levels of cTnT in the DOX-induced ACI (48 and 72 hours) and normal mice by enzyme-linked immunosorbent assay (*n* = 3). n.s. represents no significance. *P < 0.05; ***P < 0.001.

To evaluate the efficacy of the feMRI of the Art-Gd probe in vivo, we established a DOX-induced mouse ACI model, which was proven with typical features of ferroptosis (*35*). Literature has shown that there was a significant accumulation of Fe(II) and lipid peroxidase in DOX-treated cardiac tissues after 48 hours (*17*). In this respect, we used the Art-Gd probe (1.12 × 10^3^ μM Gd) to perform *T*_1_-weighted MRI 48 hours after giving DOX (10 mg/kg) intraperitoneally to healthy mice (Fig. 3A). The *T*_1_ MRI was acquired before (pre-) and after (post-) intravenous injection of the Art-Gd probe through a prefixed catheter (fig. S21). The representative *T*_1_-weighted images at pre- and postcontrast of an axial slice of the heart from each group were shown in Fig. 3 (B and C), which showed high contrast enhancement at the left ventricular (LV) cardiac tissues of the mice, especially at the interventricular septum, inferior, and inferolateral, at 60 min after intravenous injection of the Art-Gd probe, while little to no contrast enhancement was observed in the cardiac tissues of normal mice. This phenomenon was presumably attributed to the retention of the Art-Gd probe in the DOX-induced ferroptotic cardiac cells, giving rise to the reactivity-based self-enhancement in *T*_1_ MRI. As a control, Magnevist could barely enhance the contrast of LV of the mice (Fig. 3D). The semiquantitative analysis results further demonstrated that the Art-Gd probe achieved a significant change of the signal-to-noise ratio (SNR) (95.0 ± 19.1%) in the LV cardiac tissues than that of Magnevist (6.6 ± 4.5%), in which the ΔSNR = (SNR_pre_ − SNR_post_)/SNR_pre_ (***P < 0.001; Fig. 3E). We further used the Art-S-^68^Ga probe to conduct cardiac PET imaging study, which also revealed higher cardiac uptake of the Art-S-^68^Ga probe in ACI mice [2.4 ± 0.1 percentage of injected dose per gram (% ID/g)]) compared with that of normal mice (1.5 ± 0.2% ID/g) at 60 min after injection (**P = 0.0014; fig. S22). The hematoxylin and eosin (H&E) and Sirius red staining results showed the infiltration of inflammatory cells and the formation of collagen fibrils within the DOX-treated cardiac tissues (fig. S23). In addition, the immunohistochemical staining of 4-hydroxynonenal (4-HNE) suggested that the DOX-treated cardiac tissues had an elevated level of lipid peroxide compared to that of the control group. However, it is of noted that the standard echocardiography and clinical serum assays, such as the LV ejection fraction (EF), the LV fractional shortening (FS), the serum cardiac troponin T (cTnT), CK-MB, and LDH levels, did not show significant differences between normal and DOX-induced ACI mice until 72 hours [*P = 0.0106 and ***P < 0.001 (Fig. 3, F to I) and ***P < 0.001 (fig. S24)]. The H&E staining of major organs after the administration of the Art-Gd probe revealed little to no pathological abnormality, indicating the negligible systemic toxicity (fig. S25). Therefore, the feMRI strategy with the Art-Gd probe could detect the potential changes in cardiac tissues at least 24 hours earlier than the current clinical standard assays in the DOX-induced ACI mouse model.

### The feMRI in CDDP-induced AKI mouse model

**Fig. 4.**
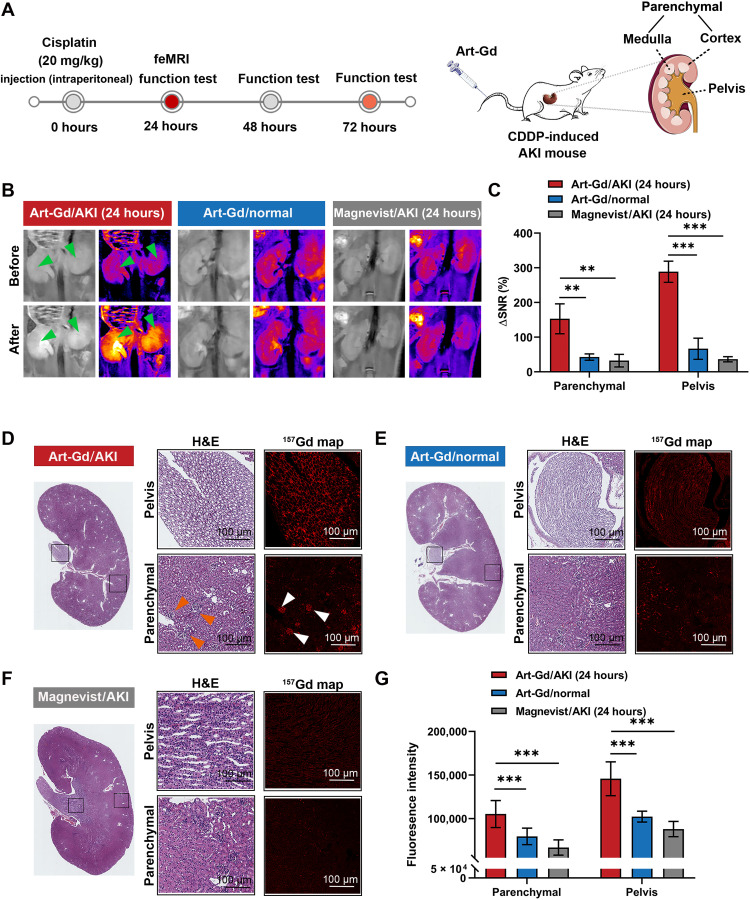
The feMRI in CDDP-induced AKI mouse model. (**A**) Scheme shows that mice were administered with cisplatin (CDDP) (20 mg/kg) by intraperitoneal injection. After 24 hours, the feMRI was performed by intravenous injection of the Art-Gd probe. The blood samples of AKI mice at 24, 48, and 72 hours were analyzed for kidney function tests. The anatomic structure of kidney shows the parenchyma (medulla plus cortex) and pelvis. (**B**) Representative *T*_1_-weighted images at pre- and postcontrast time points for different groups (*n* = 3 per group), including Art-Gd/AKI (24 hours), Art-Gd/normal, and Magnevist/AKI (24 hours). The MR images were acquired at the same in-plane axial slices through a prefixed catheter. Green arrows indicate the contrast areas. (**C**) Quantification of ΔSNR of the parenchyma and pelvis from the *T*_1_-weighted images (**P < 0.01 and ***P < 0.001). (**D** to **F**) Representative images and quantitative analysis of hematoxylin and eosin (H&E) and ^157^Gd map of kidney sections in different groups by mass spectrometry imaging (MSI), including Art-Gd/AKI (24 hours), Art-Gd/normal, and Magnevist/AKI (24 hours). White and orange arrows indicate the renal glomerulus (scale bars, 100 μm). (**G**) Quantitative analyses of the fluorescence intensity of ^157^Gd in tissues by different treatments. ***P < 0.001.

We further conducted a CDDP-induced AKI mouse model and tested the feasibility of the Art-Gd probe to detect potential ferroptotic cell death in the kidneys. Although the mechanism is not clear, literature has revealed that the dysfunction of kidney was found in mice administered with CDDP, and this situation could be alleviated by ferroptosis-related therapeutics (*19*). Before the imaging study, we first tested the changes of ferroptosis-related biomarkers, including transferrin receptor 1 (TfR1), ferritin heavy chain 1 (FTH-1), ferroportin (Fpn), and glutathione peroxidase 4 (GPX4) from mice treated with CDDP at different doses (10 or 20 mg/kg) and different time points (24 or 48 hours) by Western blotting assays (***P < 0.001; fig. S26). The expression of TfR1 and FTH-1 obviously increased at 24 hours after CDDP treatment, indicating the increased iron uptake and storage in the kidneys. By contrast, the expression levels of Fpn and GPX4 were significantly down-regulated in the CDDP-treated kidneys, indicating the limited intracellular iron efflux and the decreased anti-ferroptosis activity. Moreover, the total elemental iron level in the CDDP-treated kidneys was higher than that of the normal kidneys (fig. S27). Accordingly, we envisioned that the level of renal Fe(II) would be elevated after renal epithelial cells were exposed to CDDP, which could be detected by the Art-Gd probe in vivo. Therefore, *T*_1_-weighted MRI of the mice pretreated (24 hours) with CDDP (20 mg/kg) were studied using the Art-Gd probe (1.12 × 10^3^ μM Gd), while Magnevist was used as a control (*n* = 3) (Fig. 4A). The multislice *T*_1_-weighted images were acquired before (pre-) and after (post-) the administration of the imaging probe (figs. S28 and S29). The representative *T*_1_-weighted images at pre- and postcontrast of a coronal slice of the kidney from each group were shown in the Fig. 4B. The Art-Gd probe showed prominent *T*_1_ bright contrast in parenchyma (cortex plus medulla) and pelvis regions of kidneys of mice with CDDP treatment, whereas normal mice only showed weak contrast enhancement in the corresponding regions. As a control, Magnevist showed the negligible enhancement between the CDDP-induced AKI and normal mice in either parenchyma or pelvis regions (Fig. 4C). These phenomena indicated the abundant accumulation of intracellular Fe(II) as the epithelial cells of the kidney tubule, glomerular, and pelvis were exposed to CDDP, resulting in the reactivity-based self-enhancement in *T*_1_-weighted MRI by the Art-Gd probe. The semiquantitative analysis results suggested that the Art-Gd probe led to a 3.6- and 8.1-fold enhancement of the ΔSNR in the parenchyma (288.6 ± 30.6%) and pelvis (153.0 ± 43.0%) regions of AKI mice compared to those of normal mice. Furthermore, similar results were also observed in CDDP-induced AKI mice at 10 mg/kg after 24 hours (figs. S30 and S31). We also performed QSM for the kidney specimens at 24 hours posttreated with CDDP or saline under a single scan. The quantitative susceptibility maps showed that the change of susceptibility is unapparent between the CDDP-treated kidney [χ = 0.003 ± 0.001 parts per million (ppm)] and normal kidney (χ = 0.002 ± 0.001 ppm) (figs. S32 and S33).

To further verify the accumulation of the Art-Gd molecules in the kidneys of CDDP-induced AKI mice at 20 mg/kg after 24 hours, we used MS imaging (MSI) to evaluate of the signal intensity of Gd(III) ions from the Art-Gd probe or Magnevist in the kidney sections (Fig. 4, D and E). The results showed that the amounts of Gd(III) ions derived from the MSI intensity in the parenchyma and pelvis regions of the AKI mice were 1.5- and 2.1-fold higher than those of normal mice after 30 min, respectively. As a control, the signal intensity of the Gd(III) ions in the kidneys of Magnevist-treated AKI mice was negligible (Fig. 4F). Given that the Art-Gd probe was specific to Fe(II) ions, these results also provide evidence to link ferroptosis [Fe(II) ions] with the MRI signal (Gd). We found that the renal glomerulus had a more intense accumulation of the Art-Gd molecules compared with the renal tubules in parenchyma regions of AKI mice, probably due to the damage of the glomerular barrier by CDDP treatment that resulted in the leakage of catalytic irons from glomerular cells (Fig. 4G) (*36*). We further showed that the Gd contents in the kidney of AKI mice were 56.68 μg per gram of kidney tissues (fig. S34). Considering the density of kidney of 1.4 g·cm^−3^, this number could be converted into the concentration of 0.51 mM of Gd in the kidney of AKI mice, indicating the reliability of the observed contrast enhancement in the MRI results. The renal PET imaging study using the Art-S-^68^Ga probe showed that the AKI mice (8.0 ± 0.4% ID/g) had significantly higher renal uptake of the Art-S-^68^Ga molecules than normal mice (4.9 ± 0.3% ID/g) at 30 min after injection (*P = 0.0383 and **P = 0.0091; fig. S35). Furthermore, the proportion of 4-HNE–positive and terminal deoxynucleotidyl transferase–mediated deoxyuridine triphosphate nick end labeling–positive cells were significantly increased in the kidneys of CDDP-treated mice, indicating that the apoptosis may not be ruled out in this case (***P < 0.001; fig. S36).

**Fig. 5.**
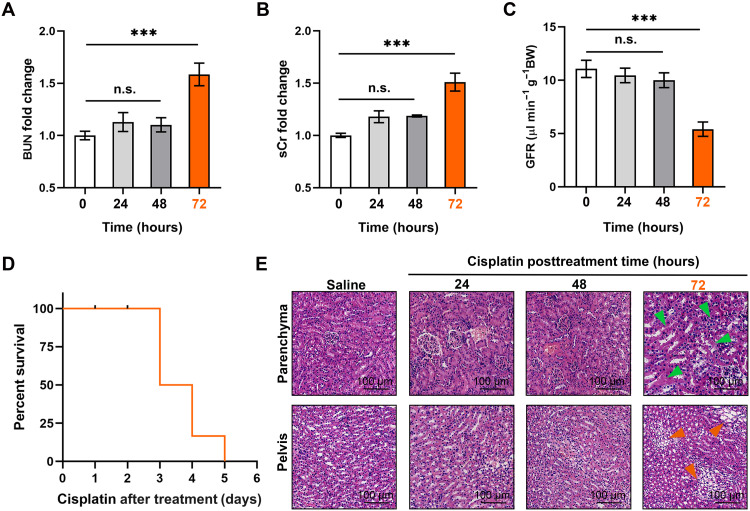
Evaluation of the kidney functions at different time points posttreated with CDDP. (**A** and **B)** The changes of blood urea nitrogen (BUN) and serum creatinine (sCr) in living mice at 0, 24, 48, and 72 hours after treatment with CDDP (20 mg/kg) (*n* = 3). (**C**) The glomerular filtration rate (GFR) of living mice at 0, 24, 48, and 72 hours after treatment with CDDP (20 mg/kg), measured by the standard fluorescein isothiocyanate (FITC)-inulin assay (*n* = 3). (**D**) The mouse survival rates for the CDDP-induced AKI mice. (**E**) Representative photomicrographs of H&E staining from mice after treatment of saline or CDDP [20 mg/kg body weight (BW)] at 0, 24, 48, and 72 hours. Green arrows indicate the damaged tubules, and orange arrows indicate the infiltration of multifocal foam cells (scale bars, 100 μm). n.s. represents no significance. ***P < 0.001.

To further investigate whether the feMRI was capable of early detection of CDDP-induced AKI, we compared the MRI results with the changes of renal biomarkers typically used in the clinical standards. The levels of blood urea nitrogen (BUN) and sCr as well as the glomerular filtration rate (GFR) were evaluated in the normal and AKI mice at different time points after treatment with CDDP (24, 48, and 72 hours). Statistically significant increases in BUN and sCr were observed only at 72 hours after treatment of CDDP, which were 1.7- and 1.5-fold higher than those of normal mice, respectively (***P < 0.001; Fig. 5, A and B). The increased BUN and sCr levels were attributed to a decline of the kidney functions, which was further confirmed by the observation of around 50% decrease in GFR at 72 hours after treatment of CDDP (Fig. 5C). Furthermore, we also measured other conventional AKI biomarkers, including NGAL and KIM-1, which showed the statistically significant change at 48 and 72 hours after treatment with CDDP, respectively (***P < 0.001; fig. S37). Notably, there were a half of the mice that died within 72 hours after treatment with CDDP (Fig. 5D). The H&E staining results showed damaged tubules and infiltration of multifocal foam cells in parenchyma and pelvis morphology at 72 hours after treatment of CDDP, respectively (Fig. 5E). These results implied that the Art-Gd probe could detect the early stage of the AKI at 24 hours posttreated with CDDP through the feMRI strategy, while the current clinical standards did not show changes until 72 hours after treatment under the same conditions.

### Evaluating the therapeutic efficacy in AKI by the feMRI

**Fig. 6.**
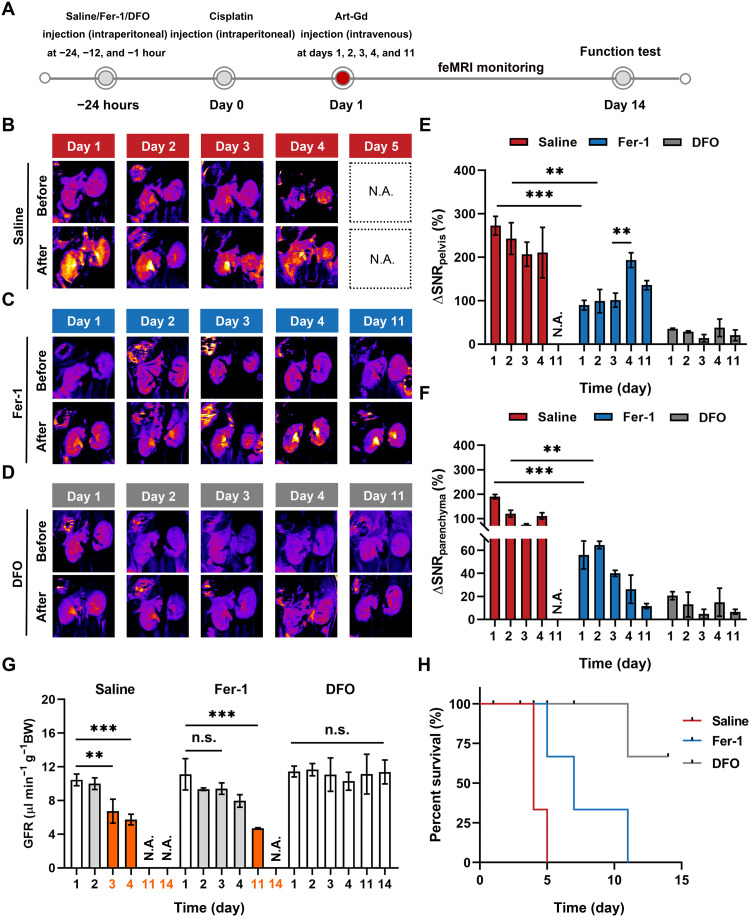
The feMRI monitoring of the therapeutic efficacy in CDDP-induced AKI mouse model. (**A**) Scheme shows the experimental procedure. Mice were treated with intraperitoneal injection of CDDP (20 mg/kg). Saline, ferrostatin-1 (Fer-1), or desferrioxamine (DFO) were administered at −24, −12, and −1 hour before CDDP treatment, respectively. *T*_1_-weighted MRI was conducted to acquire pre- and postcontrast *T*_1_ MRI before and after intravenous injection of the Art-Gd probe (1.12 × 10^3^ μM Gd) on days 1, 2, 3, 4, and 11 after treatment with CDDP. On day 14, mice were euthanized to collect blood and tissue samples for kidney function tests. (**B** to **D**) Representative *T*_1_-weighted images (pseudo-color) of mouse kidneys from different groups at pre- and postcontrast points were acquired on days 1, 2, 3, 4, and 11 after treatment with CDDP. N.A. means not available due to death of the mouse group. (**E** and **F**) Quantification of ΔSNR of the parenchyma and pelvis for the *T*_1_-weighted images from different groups (*n* = 4 or 5 per group; **P < 0.01; ***P < 0.001). (**G**) The GFR of living mice after treatment with saline, Fer-1, and DFO on days 1, 2, 3, 5, and 11 following CDDP treatment, as measured by the standard FITC-inulin assay (*n* = 3). (**H**) The mouse survival rates of AKI mice were recorded up to 14 days for different treatments. n.s. represents no significance. **P < 0.01; ***P < 0.001.

Evidences indicated that inhibiting ferroptosis could serve as a renoprotective strategy for the anticancer drug–induced AKI (*21*). However, the in vivo evaluation of the therapeutic efficacy by targeting ferroptosis mechanism during the treatment period is less studied. Given the feasibility of the feMRI in vivo, we used the Art-Gd probe to assess the therapeutic efficacy of two ferroptosis inhibitors (Fer-1 and DFO) in CDDP-treated mice. Before CDDP treatment, three mouse groups were intraperitoneally injected with saline, Fer-1, or DFO with their respective effective doses according to literature (Fig. 6A) (*19*). Subsequently, the Art-Gd probe was intravenously injected into mice of the different treatment groups on days 1, 2, 3, 4, and 11 posttreated with CDDP and followed by *T*_1_-weighted MRI acquisition (Fig. 6, B to D, and fig. S38). The results showed that the kidneys from saline-treated mice had strong contrast with ΔSNR_pelvis_ and ΔSNR_parenchyma_ of 210.6 ± 48.2 and 110.6 ± 12.9% at day 4 after treatment of CDDP, respectively (Fig. 6, E and F), implying the sustained damage of CDDP in both the kidney parenchyma and pelvis regions. By contrast, the significantly lower contrast was observed within the first 2 days of Fer-1 treatment with ΔSNR_pelvis_ of 89.7 ± 11.0 and 99.0 ± 26.9% (***P = 0.0002 and **P = 0.0053) and ΔSNR_parenchyma_ of 56.0 ± 12.2 and 64.7 ± 3.2% (***P = 0.0001 and **P = 0.0030) on days 1 and 2, respectively. However, the ΔSNR_pelvis_ of Fer-1–pretreated mice had a significant increase after 3 days (**P = 0.0030). Very low contrast in both the parenchyma and pelvis regions were observed in the DFO–pretreated mouse group, with overall ΔSNR_pelvis_ and ΔSNR_parenchyma_ at around 30 and 10% or less, respectively. These results demonstrated that DFO could provide the long-term protection from the nephrotoxicity of CDDP, whereas Fer-1 under the equivalent condition was less effective, especially to the pelvis region of kidneys. The different therapeutic efficacy of the two ferroptosis inhibitors is possibly due to the different mechanisms of action, either by blocking lipid peroxidation or depleting iron ions. To further validate the therapeutic effect of Fer-1 and DFO, the GFR, BUN, and sCr levels from each group were measured (Fig. 6G). On day 3 after different treatments, the GFR level of mice pretreated with saline reduced by around 40% (from 10.4 ± 0.7 to 6.7 ± 1.4 μl min^−1^ g^−1^), while the GFR level of mice pretreated with Fer-1 slightly decreased from 11.1 ± 1.9 to 9.4 ± 0.7 μl min^−1^ g^−1^, which were within the normal range (*37*). However, a significantly decreased GFR level to 4.7 ± 0.1 μl min^−1^ g^−1^ (**P = 0.0040) was found in the Fer-1–pretreated mice on day 11 after CDDP treatment. Note that the GFR level of DFO–pretreated mice remained within the normal range during the treatment period. Similar trends were observed for the BUN and sCr levels of the different treatment groups (fig. S39). Furthermore, the kidney tissues from each group at their respective treatment end points were collected for H&E staining. Damaged tubules and pelvis were observed in the kidney sections from mice pretreated with saline on day 4 (all the mice died on day 4 or 5). In Fer-1–pretreated mice, an alleviated damage of tubules and pelvis was observed on day 3, which unfortunately advanced to a more severe stage afterwards. No observable damage on kidney tissues was found within 14 days for DFO–pretreated mice (fig. S40). The prolonged survival rate of mice pretreated with DFO further highlighted the potent renoprotection effect when compared with that of saline- and Fer-1–pretreated mice (Fig. 6H). Collectively, the Art-Gd probe could serve as an efficient feMRI contrast agent for early and noninvasively evaluation of the therapeutic efficacy of ferroptosis-targeted agents in CDDP-induced AKI mouse model.

## DISCUSSION

Over the last decade, ferroptosis has gained great momentum in refreshing the understanding of a variety of common diseases including tumors (*38*), organ dysfunctions (*39*), and neurodegenerative disorders (*40*). In general, most ferroptosis-related diseases are also linked to the activation of sterile inflammation, which is known as a part of innate immunity in response to physiochemical insults (*41*, *42*). Although it is still controversial whether cell death is the reason or the outcome of the inflammatory process, necrosis and/or apoptosis are often recognized as indicators of the disease advancement. In this regard, because of the unique features of labile iron pool and lipid peroxidation, ferroptosis may serve as an alternative mechanism to the etiology and the treatment deployment of those inflammatory diseases. This hypothesis was also examined in the clinical trials that some ferroptosis inhibitors (e.g., DFO and vitamin E) showed possible clinical benefits in Alzheimer’s disease treatment (*43*, *44*). For anticancer drug–induced ACI and AKI, both the abnormality of iron metabolism and the alleviation effect by ferroptosis-targeting therapeutics were evidenced in multiple mouse models, which collectively linked the anticancer drug–induced ACI and AKI with ferroptosis (*14*, *16*, *17*). Therefore, we established our study on the hypothesis that molecular imaging of ferroptosis may facilitate the deconvolution of the anticancer drug–induced ACI/AKI. However, molecular imaging approach to detect ferroptosis in the ACI/AKI in vivo is rarely reported, which dampens the rationality and enthusiasm to propel the development of theranostics targeting ferroptosis. To this end, we further proposed that the intracellular accumulation of Fe(II) could serve as a vivid chemical target to mirror ferroptosis considering the meticulous iron homeostasis in healthy body.

Different from traditional approaches targeting macromolecules through ligand-receptor interactions, targeting Fe(II) evokes a prototypical shift to the development of molecular imaging approaches in vivo. MRI is a widely used diagnostic tool in the clinic because of the noninvasiveness, the anatomical feature, and the high sensitivity to soft tissues (*45*). In addition, contrast-enhanced MRI with augmented sensitivity and specificity holds great potential in precision diagnosis of specific biological targets (*46*). The multiple contrast mechanisms provide great flexibility to the designing of various MRI probes for different scenarios (*47*). In this work, we designed an Art-Gd probe for targeted ferroptosis MRI based on the radical formation reaction between the artemisinin molecules and the chemically reactive Fe(II). The produced radicals from the artemisinin motif allow the Art-Gd molecules to attach onto their surrounding biological substrates such as proteins and lipid membranes. As a result, the reactivity-based self-enhancement mechanism of the Art-Gd probe confers two important advantages for the feMRI: (i) the enhanced retention effect in the targeted tissues and (ii) the enhanced *T*_1_ contrast efficiency due to the slow tumbling effect of the entity. Previous studies have reported the design of fluorescence and PET imaging Fe(II) probes based on the fabrication of a TRX moiety through a multistep synthetic chemistry (*28*, *29*). To the best of our knowledge, we present a simple chemistry of designing artemisinin-based molecular probes for MRI of Fe(II) through elaborating the contrast-enhancement mechanism in MRI. We anticipated that the simple but robust design of molecular probes may promise to the translational study in the future.

By taking advantages of the Art-Gd probe, the feMRI was able to detect the abnormal intracellular level of Fe(II) in the cardiac and renal cells of the anticancer drug–induced ACI and AKI mice. The feMRI results were at least 24 and 48 hours earlier than the commonly used clinical indexes of cTn and sCr for the ACI and AKI, respectively. These results indicated that the change of Fe(II) in situ occurred much earlier than that of serum biomarkers excreted from the primary tissues, facilitating early diagnosis of tissue-specific diseases. Furthermore, the feMRI provided a robust means to differentiate the therapeutic efficacy of two ferroptosis inhibitors (i.e., blocking lipid peroxidation or depleting iron ions) in CDDP-induced AKI. On this occasion, we anticipate that this approach could be used for the evaluation of the cardiac and kidney functions in patients with cancer during chemotherapy in a real-time manner, allowing for the timely adjustment of the therapy deployment and ultimately improving the treatment outcomes. Last, but not least, the Art-Gd probe may be amenable to the feMRI of other ferroptosis-related diseases, such as neurodegenerative disorders, ischemia-reperfusion injury, and so on.

To summarize, we report a reactivity-based Art-Gd molecular probe for the early evaluation of anticancer drug–induced ACI/AKI through the feMRI strategy. The Art-Gd probe was able to show contrast in MRI at least 24 and 48 hours earlier than the standard clinical assays for assessing ACI and AKI, respectively. Moreover, the feMRI was able to provide molecular imaging evidence of two different mechanisms of action for ferroptosis-targeted agents, either by blocking lipid peroxidation or depleting iron ions. The Art-Gd probe may serve as a general tool to study ferroptosis process in vivo by the feMRI strategy, which sheds light on molecular imaging and drug development for the theranostics of a variety of ferroptosis-related diseases.

## MATERIALS AND METHODS

### Materials

DHA, 3-mercaptopropionic acid, *N*-hydroxysuccinimide (NHS), succinic anhydride, 4-dimethylaminopyridine (DMAP), 1-ethyl-3-(3-dimethylaminopropyl)carbodiimide hydrochloride (EDCI), and dicyclohexylcarbodiimide were purchased from Sigma-Aldrich. DOTA-NH_2_ was purchased from Confluore Biological Technology Co. Ltd. Fer-1 and DOX hydrochloride were purchased from MedChemExpress. CDDP and DFO were purchased from Energy Chemical. Anti–4-HNE (bs-6313R) was purchased from Bioss. Anti-GPX4 (ab125066) was purchased from Abcam. Anti-TfR1 (BA0462) was purchased from Boster. Anti–FTH-1 (no. 3998) was purchased from Cell Signaling Technology. Slc40A1 (Fpn, AB-23233-A) was purchased from Alpha Diagnostics. β-Actin (8H10D10) was purchased from Cell Signaling Technology. FerroOrange was purchased from Maokang Biotechnology. The creatinine assay kit and BUN assay kit were purchased from Ponstar Biotech Co. Ltd.

### Synthesis of Art-S-COOH

A flame-dried two-neck round-bottom flask was charged with DHA (200 mg, 0.70 mmol, 1.0 eq) and anhydrous dichloromethane (10 ml). 3-Mercaptopropionic acid (67 μl, 0.77 mmol, 1.1 eq) was added and stirred for 10 min at 50°C under N_2_ atmosphere. Boron trifluoride diethyl etherate (89 μl, 0.70 mmol, 1.0 eq) was added dropwise, and the reaction was stirred for 30 min at 0°C under argon. The reaction was quenched with water (5 ml) and extracted with dichloromethane (3 × 10 ml). The organic layers were combined, dried over Na_2_SO_4_, and concentrated under a vacuum to afford Art-S-COOH (157 mg, 60%) as a white solid. ^1^H NMR (400 MHz, CDCl_3_): δ 5.34 (s, 1H), 4.62 (d, *J* = 10.8 Hz, 1H), 3.12 (dt, *J* = 12.3, 5.8 Hz, 1H), 3.01 to 2.76 (m, 3H), 2.70 (ddd, *J* = 11.1, 7.5, 4.5 Hz, 1H), 2.41 (td, *J* = 13.9, 4.0 Hz, 1H), 2.06 (d, *J* = 15.1 Hz, 1H), 1.98 to 1.88 (m, 1H), 1.76 (s, 2H), 1.69 to 1.60 (m, 1H), 1.47 (d, *J* = 4.4 Hz, 4H), 1.36 to 1.25 (m, 2H), 1.18 to 1.06 (m, 1H), and 1.04 to 0.97 (m, 6H). Electrospray ionization MS (ESI-MS): (*m*/*z*): calcd.: 372.1604; found: [M–H]^−^, 371.1265; [2 M–H]^−^, 743.2687.

### Synthesis of Art-O-COOH

A flame-dried two-neck round-bottom flask was charged with DHA (200 mg, 0.70 mmol, 1.0 eq) and anhydrous dichloromethane (10 ml). Succinic anhydride (70 mg, 0.70 mmol, 1.0 eq), DMAP (78 mg, 0.70 mmol, 1.0 eq), and EDCI (267 mg, 1.40 mmol, 2.0 eq) at 0°C were added and stirred for 4 hours. The reaction mixture was quenched with water (5 ml) and extracted with dichloromethane (3 × 10 ml). The organic layers were combined, dried over Na_2_SO_4_, and concentrated under a vacuum to afford Art-O-COOH (177 mg, 61%) as a white solid. ^1^H NMR (400 MHz, CDCl_3_): δ 5.80 (d, *J* = 9.9 Hz, 1H), 5.44 (s, 1H), 2.79 to 2.64 (m, 4H), 2.61 to 2.53 (m, 1H), 2.38 (td, *J* = 14.1, 3.9 Hz, 1H), 2.07 to 2.00 (m, 1H), 1.93 to 1.85 (m, 1H), 1.81 to 1.69 (m, 2H), 1.66 to 1.59 (m, 1H), 1.55 to 1.46 (m, 1H), 1.43 (s, 3H), 1.41 to 1.36 (m, 2H), 1.30 (dd, *J* = 10.6, 4.7 Hz, 1H), 1.03 (dd, *J* = 20.6, 8.0 Hz, 1H), 0.96 (d, *J* = 5.9 Hz, 3H), and 0.85 (d, *J* = 7.1 Hz, 3H).

### Synthesis of Art-S-DOTA

A round-bottom flask was charged with Art-S-COOH (500 mg, 1.34 mmol, 1.0 eq), dicyclohexylcarbodiimide (554 mg, 2.68 mmol, 2.0 eq), and NHS (232 mg, 2.12 mmol, 1.5 eq); and anhydrous dichloromethane (10 ml) was stirred at 0°C for 1 hour. After overnight stirring at room temperature, the reaction mixture was filtered through a bed of celite. The celite was washed with additional dichloromethane (3 × 20 ml). The filtrate was concentrated to give Art-S–NHS ester as a white solid. A round-bottom flask was charged with Art-S-NHS ester (100 mg, 0.21 mmol, 1.0 eq), DOTA-NH_2_ (110 mg, 0.21 mmol, 1.0 eq), *N*,*N*-diisopropylethylamine (250 μl, 0.978 mmol, 10.0 eq), and dry dimethyl sulfoxide (DMSO) (6 ml). The resulting mixture was stirred overnight and purified by reverse phase HPLC (Thermo Fisher Scientific C18 column) held at eluting with a gradient of 20 to 95% CH_3_CN (0.1% CF_3_COOH) in water (0.1% CF_3_COOH) over 40 min, *t*_r_ = 13.9 min. This gave about 80 mg of the product as a white solid after lyophilization. ^1^H NMR (400 MHz, D_2_O): δ 5.46 (d, *J* = 2.7 Hz, 1H), 3.76 (s, 7H), 3.41 to 3.19 (m, 25H), 2.91 (dt, *J* = 34.1, 8.8 Hz, 4H), 2.58 (q, *J* = 7.0, 6.6 Hz, 3H), 2.32 to 2.19 (m, 2H), 2.07 (d, *J* = 15.4 Hz, 1H), 1.97 to 1.84 (m, 1H), 1.66 (q, *J* = 14.4, 12.2 Hz, 5H), and 1.36 (d, *J* = 2.8 Hz, 8H). ESI-MS (*m*/*z*): calcd.: 800.3990; found: [M–H]^−^, 799.3886.

### Synthesis of Art-O-DOTA

A round-bottom flask was charged with Art-O-COOH (200 mg, 0.52 mmol, 1.0 eq), dicyclohexylcarbodiimide (214 mg, 1.04 mmol, 2.0 eq), and NHS (89.7 mg, 0.78 mmol, 1.5 eq); and anhydrous dichloromethane (5 ml) was stirred at 0°C for 1 hour. After overnight stirring at room temperature, the reaction mixture was filtered through a bed of celite. The celite was washed with additional dichloromethane (3 × 20 ml). The filtrate was concentrated to give Art-O-NHS ester as a white solid. A round-bottom flask was charged with Art-O-NHS ester (100 mg, 0.21 mmol, 1.0 eq), DOTA-NH_2_ (110 mg, 0.21 mmol, 1.0 eq), N,N-Diisopropylethylamine (DIPEA) (250 μl, 0.978 mmol, 10.0 eq), and dry DMSO (6 ml). The resulting mixture was stirred overnight and purified by reverse phase HPLC (Thermo Fisher Scientific C18 column) held at eluting with a gradient of 20 to 95% CH_3_CN (0.1% CF_3_COOH) in water (0.1% CF_3_COOH) over 40 min, *t*_r_ = 12.9 min. This gave about 40 mg of the product as a white solid after lyophilization. ^1^H NMR (400 MHz, DMSO-*d*_6_): δ 5.56 (s, 1H), 3.58 (s, 21H), 3.11 (s, 10H), 2.61 (t, *J* = 7.0 Hz, 2H), 2.17 (d, *J* = 10.6 Hz, 1H), 1.97 (s, 1H), 1.81 (s, 1H), 1.76 (s, 1H), 1.68 to 1.39 (m, 5H), 1.29 (s, 4H), 0.89 (d, *J* = 6.2 Hz, 3H), and 0.77 (d, *J* = 7.0 Hz, 3H). ESI-MS (*m*/*z*): calcd.: 812.4168; found: [M–H]^−^, 811.2917; [2 M–H]^−^, 1623.6786.

### Synthesis of Art-Gd

Art-S-DOTA (65 mg, 0.1 mmol) was dissolved in 10 ml of deionized H_2_O, and GdCl_3_·6H_2_O (194.7 mg, 0.3 mmol, 3.0 eq) was added. The pH was adjusted to ~4 with 0.1 M NaOH, and the resulting mixture was stirred overnight at 50°C. After lyophilization, the obtained white solid was dissolved in CH_3_CN and further purified by reverse phase HPLC (Thermo Fisher Scientific C18 column) held at eluting with a gradient of 20 to 95% CH_3_CN (0.1% CF_3_COOH) in water (0.1% CF_3_COOH) over 40 min. ESI + MS (*m*/*z*): calcd.: 952.3990 to 957.3990; found: [M+H]^+^, 953.5249, 954.5231, 955.5222, 956.5219, 957.5220, and 958.5227.

### Cell culture and animal models

Murine cardiac H9c2 and human embryonic kidney HEK293T cells were acquired from the American Type Culture Collection and cultured in Dulbecco’s modified Eagle’s medium containing 10% heat-inactivated fetal bovine serum and supplemented to a final concentration with l-glutamine (2 mM), penicillin (50 U/ml), and streptomycin (50 μg/ml). All animal experiments were carried out in accordance with Guide for the Care and Use of Laboratory Animals, approved by the Ethics Committee of the Xiamen University. Six- to eight-week-old female C57BL/6 mice were ordered from the Xiamen University Animal Center. To establish an ACI mouse model, C57BL/6 mice were treated with DOX (10 mg/kg body weight, intraperitoneal injection). To establish AKI mouse model, C57BL/6 mice were treated with CDDP (10 or 20 mg/kg body weight, intraperitoneal injection) and water deprivation for 24 hours before treatment.

### Western blotting assay

Kidney tissues from AKI mice were lysed using radioimmunoprecipitation assay lysis buffer (50 mM tris, 150 mM NaCl, 1% Triton X-100, 1% sodium deoxycholate, and 0.1% SDS) and centrifuged at 10,000 revolutions per minute (rpm) for 5 min at 4°C. SDS–polyacrylamide gel electrophoresis was operated with 10 μg of proteins per well at a voltage of 100 V for 90 min. The proteins were transferred to a polyvinylidene difluoride (PVDF) membrane using wet transfer mode (Bio-Rad) at 260 mA for 50 min. Membranes were incubated in primary antibodies overnight at 4°C and then washed with tris-buffered saline (TBS) containing 0.05% Tween 20 for five times each with 5 min. The PVDF membranes were then individually incubated with the following antibodies at the stated dilutions overnight at 4°C: anti–FTH-1, anti-GPX4, anti-Fpn, anti-TfR1, and anti–β-actin. The membranes were washed with TBST buffer [137 mM NaCl, 2.7 mM KCl, 16.5 mM tris (pH 7.4), containing 0.1% Tween 20] and incubated with secondary antibodies at 22°C for 60 min. After another wash with TBST, the detection of proteins was performed using enhanced chemiluminescence, and the results were analyzed and quantified by ImageJ (v1.8.0).

### MRI measurements

The MRI phantom study was conducted on a 9.4-T scanner (Bruker) using *T*_1_ and *T*_2_ mapping sequences, rapid acquisition with relaxation enhancement with variable repetition time (RARE-VTR), and multislice multiecho, respectively. The phantom samples with different concentrations of Art-Gd, Art-Gd + Fe(II)/Heme, Art-Gd + BSA, Art-Gd + Fe(II)/Heme + BSA, Fe(II)/Heme, and FeCl_2_ were prepared and studied by *T*_1_ and *T*_2_ MRI in parallel for comparison purposes. The *T*_1_ phantom MRI acquisition used the following parameters: repetition time (TR) = 1000 ms; echo time (TE) = 8.5 ms; inversion times = 5500, 3000, 1500, 800, 400, and 327.103 ms; 256 by 256 matrices. The *T*_2_ phantom MRI acquisition used the following parameters: TR = 2500 ms; TE = 33 ms; inversion times = 11, 22, 33, 44, 55, 66, 77, 88, 99, 110, 121, 143, 154, and 165 ms; 256 by 256 matrices.

### EPR measurements

Detection of carbon-centered radicals of the Art-Gd probe was performed using DMPO as the spin trapping agent. In experiments with DMPO, Art-Gd (1 mM), FeCl_2_ (1 mM), and DMPO (200 mM) were mixed (ACN/H_2_O = 1:1). The samples were measured after vortex immediately, and 50 μl of aliquots were aspirated in glass capillaries and transferred in the resonator of the EPR spectrometer. The measurements were performed in a Wildman Suprasil/aqueous quartz-ware flat cell with a Bruker EMXplus-9.5/12 EPR spectrometer using the following parameters: microwave power, 20 mW; modulation amplitude, 1.0 G; and modulation frequency, 100 kHz.

### The feMRI in DOX-induced ACI or CDDP-induced AKI mouse model

The in vivo MRI of DOX-induced ACI or CDDP-induced AKI mice was conducted by a 9.4-T scanner (Bruker). We used a prefixed catheter in the mouse tail vein to acquire pre- and postcontrast *T*_1_. After sequential scanning of precontrast *T*_1_-weighted MRI using the same in-plane geometries, we injected the Art-Gd probe (1.12 × 10^3^ μM Gd) intravenously from outside the scanner, while the mouse was kept anesthetized and left steady. Then, we acquired the postcontrast *T*_1_-weighted MRI at different postinjection times through the same sequence parameters. The imaging sequence for *T*_1_-weighted MRI was RARE-VTR pulse using the following parameters: TE = 8.5 ms; effective TE = 8.5 ms; rare factor = 4; number of experiments = 6; multiple repetition time = 5500, 3000, 1500, 800, 400, and 327.103 ms; number of averages = 1; number of repetitions = 1; matrix = 256 by 256; and scan time = 9 min, 13 s, and 300 ms.

### Evaluating the therapeutic efficacy in CDDP-induced AKI mice

Saline, Fer-1, and DFO with their respective effective doses according to literature *(**19**)* were intraperitoneally injected −24, −12, and −1 hour before CDDP injection, respectively. Subsequently, *T*_1_-weighted MRI was conducted to acquire pre- and postcontrast *T*_1_ before and after intravenous injection of the Art-Gd probe (1.12 × 10^3^ μM Gd) at *t* = 1, 2, 3, 4, and 11 days posttreated with CDDP (20 mg/kg). The blood samples of AKI mice at different time points posttreated with CDDP were collected for analysis of kidney function. At the treatment end point, kidney tissues from each group were collected for histological examination.

### Quantitative susceptibility mapping

Data were acquired using three-dimensional gradient-echo (GRE) sequence on a 9.4-T scanner (Bruker) with the following acquisition parameters: voxel size = 0.187 mm^3^ isotropic, field of view = 24 by 24 by 15 mm^3^, TE_1_/spacing/TE_8_ = 1.884/2/15.884 ms, TR = 80 ms, and flip angle = 20°. The data processing for QSM were all implemented using susceptibility tensor imaging (STI) Suite toolbox (https://people.eecs.berkeley.edu/~chunlei.liu/software.html). Specifically, the raw phase was unwrapped by Laplacian-based phase unwrapping method (*48*). Then, the tissue phase was obtained by removing the background phase using variable-kernel-Sophisticated harmonic artifact reduction for phase data (V-SHARP) (*49*). To obtain the local field map, the tissue phases from different echoes were normalized by 2πγ·TE·*B*_0_ and averaged together, where γ is the gyromagnetic ratio, TE is the echo time, and *B*_0_ is the main magnetic field strength. Last, the improved sparse linear equation and least squares (iLSQR) algorithm (*50*) was used to perform dipole inversion to obtain the susceptibility map. Note that the mask used in data processing was generated manually.

### Determination of GFR

Fluorescein isothiocyanate (FITC)–inulin (150 mg) was dissolved in 0.9% NaCl (3 ml) at 75°C and dialyzed in 0.9% NaCl (1000 ml) at 25°C for 24 hours. Dialyzed FITC-inulin (3.74 μl/g body weight) was intravenously injected into living C57BL/6 mice at *t* = 8, 12, 16, 24, and 48 hours after treatment with CDDP-treated (20 mg/kg body weight) or saline-treated (0.2 ml) mice. Blood (approximately 20 μl) was collected via orbital at 3, 7, 10, 15, 35, 55, and 75 min after injection of FITC-inulin and then centrifuged for 20 min at 3500 rpm. The serum sample (10 μl) was diluted with Hepes buffer (40 μl, 500 mM, pH 7.4), and fluorescence was measured using a SpectraMax with excitation at 485 nm and emission at 538 nm. Serum fluorescence data were presented as a two-component exponential decay curve using nonlinear regression. GFR was calculated according to the equation: GFR = *I*/(*A*/α + *B*/β), where *I* is the amount of FITC-inulin delivered by the bolus injection, *A* and *B* are the *y* intercept values of the two decay rates, and α and β are the decay constants for the distribution and elimination phases, respectively.

### Blood analysis

Blood was collected from the eye socket in living C57BL/6 mice under isoflurane anesthesia at *t* = 24, 48, and 72 hours after treatment with CDDP-treated (20 mg/kg body weight), DOX-treated (10 mg/kg body weight), and saline-treated (0.2 ml) treated mice. The collected blood samples were centrifuged for 20 min at 3500 rpm. The BUN, sCr, cTn, LDH, CK-MB, KIM-1, and NGAL were determined using commercial kits according to the manufacturer’s protocol.

## Notes

View/request a protocol for this paper from *Bio-protocol*.
