# Supplementary material for: Ferroptosis MRI for early detection of anticancer drug–induced acute cardiac/kidney injuries
Source: Sci Adv. 2023 Mar 8;9(10):eadd8539. doi: 10.1126/sciadv.add8539 (PMC9995079; doi:10.1126/sciadv.add8539)
Supplement: Supplementary file 1 — Figs. S1 to S40 Table S1 [file sciadv.add8539_sm.pdf]

Science Advances
AAAS

Supplementary Materials for

Ferroptosis MRI for early detection of anticancer drug–induced acute cardiac/kidney injuries

Fantian Zeng *et al.*

Corresponding author: Zijian Zhou, zhouz@xmu.edu.cn; Xiaoyuan Chen, chen.shawn@nus.edu.sg

Sci. Adv. **9**, eadd8539 (2023)
DOI: 10.1126/sciadv.add8539

This PDF file includes:

Figs. S1 to S40
Table S1

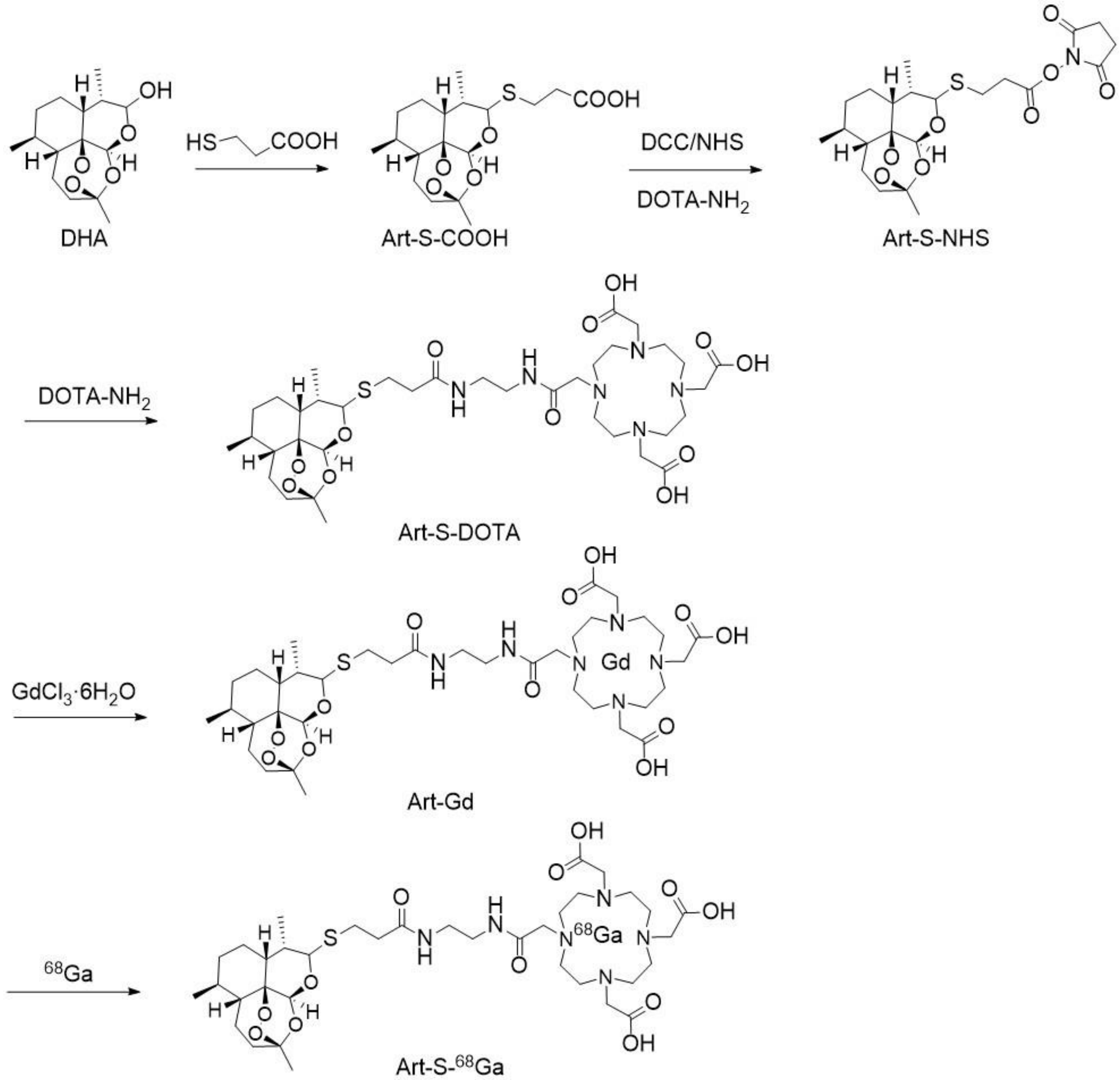

Fig. S1. The synthetic route of the Art-Gd/Art-S-^{68}Ga probe. DCC: 1, 3-dicyclohexylcarbodiimide; NHS: N-hydroxysuccinimide, DOTA-NH_2: 2,2’,2’’-(10-(4-((2-aminoethyl)amino)-1-carboxy-4-oxobutyl)-1,4,7,10-tetraazacyclododecane-1,4,7-triyl)triacetic acid.

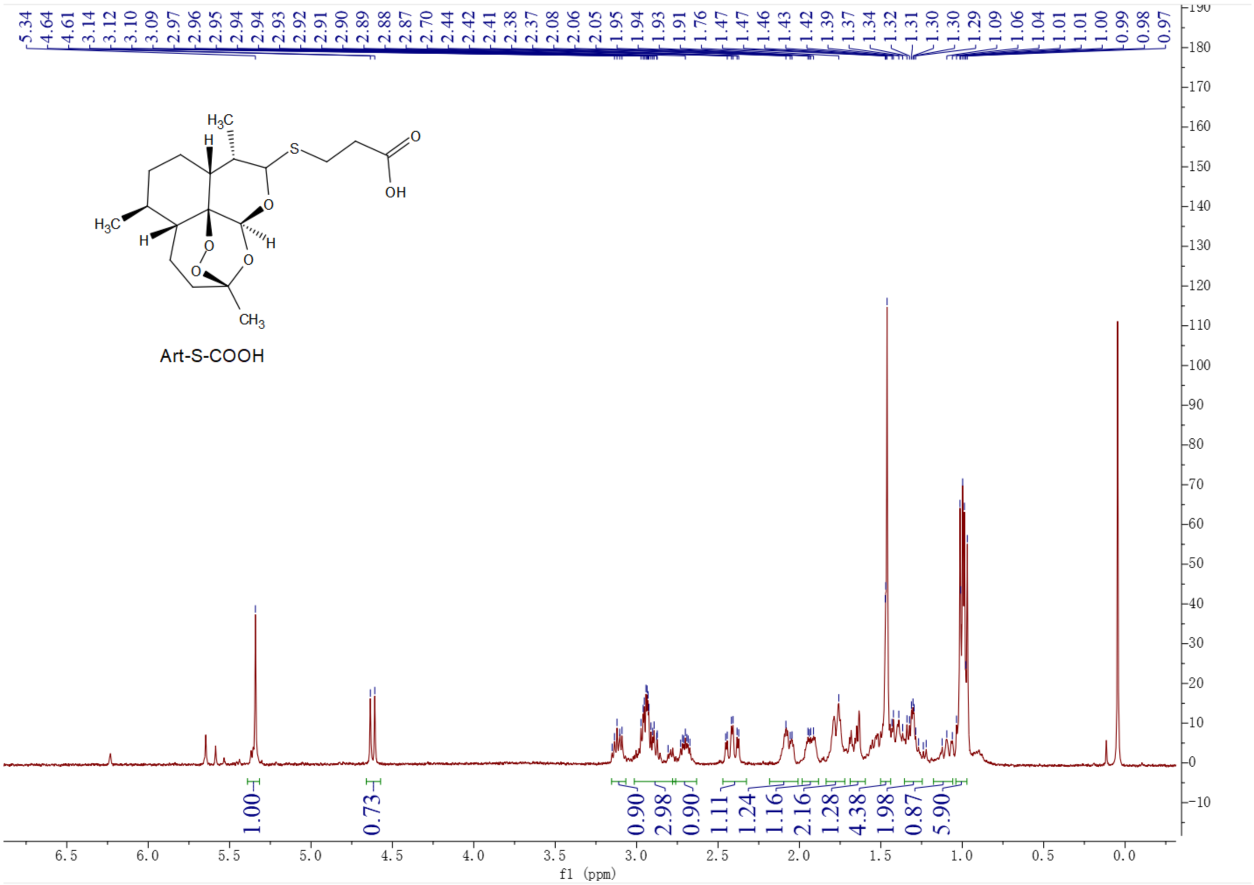

Fig. S2. ^{1}H NMR spectrum of Art-S-COOH. ^{1}H NMR (400 MHz, $CDCl_3$): δ 5.34 (s, 1H), 4.62 (d, J = 10.8 Hz, 1H), 3.12 (dt, J = 12.3, 5.8 Hz, 1H), 3.01-2.76 (m, 3H), 2.70 (ddd, J = 11.1, 7.5, 4.5 Hz, 1H), 2.41 (td, J = 13.9, 4.0 Hz, 1H), 2.06 (d, J = 15.1 Hz, 1H), 1.98-1.88 (m, 1H), 1.76 (s, 2H), 1.69-1.60 (m, 1H), 1.47 (d, J = 4.4 Hz, 4H), 1.36-1.25 (m, 2H), 1.18-1.06 (m, 1H), 1.04-0.97 (m, 6H).

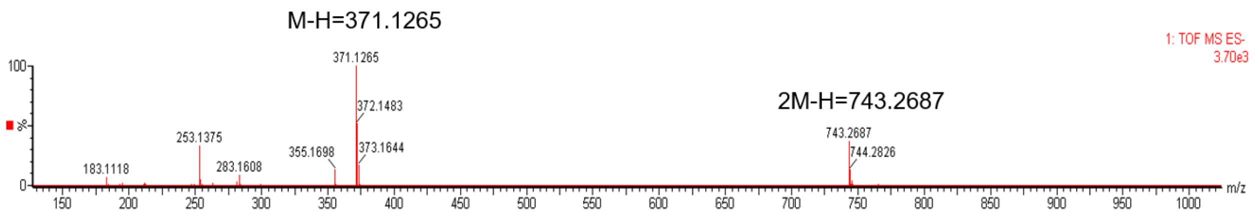

Fig. S3. MS spectrum of Art-S-COOH. ESI-MS (m/z): calcd: 372.1604, found $[M-H]^-$:371.1265; $[2M-H]^-$: 743.2687.

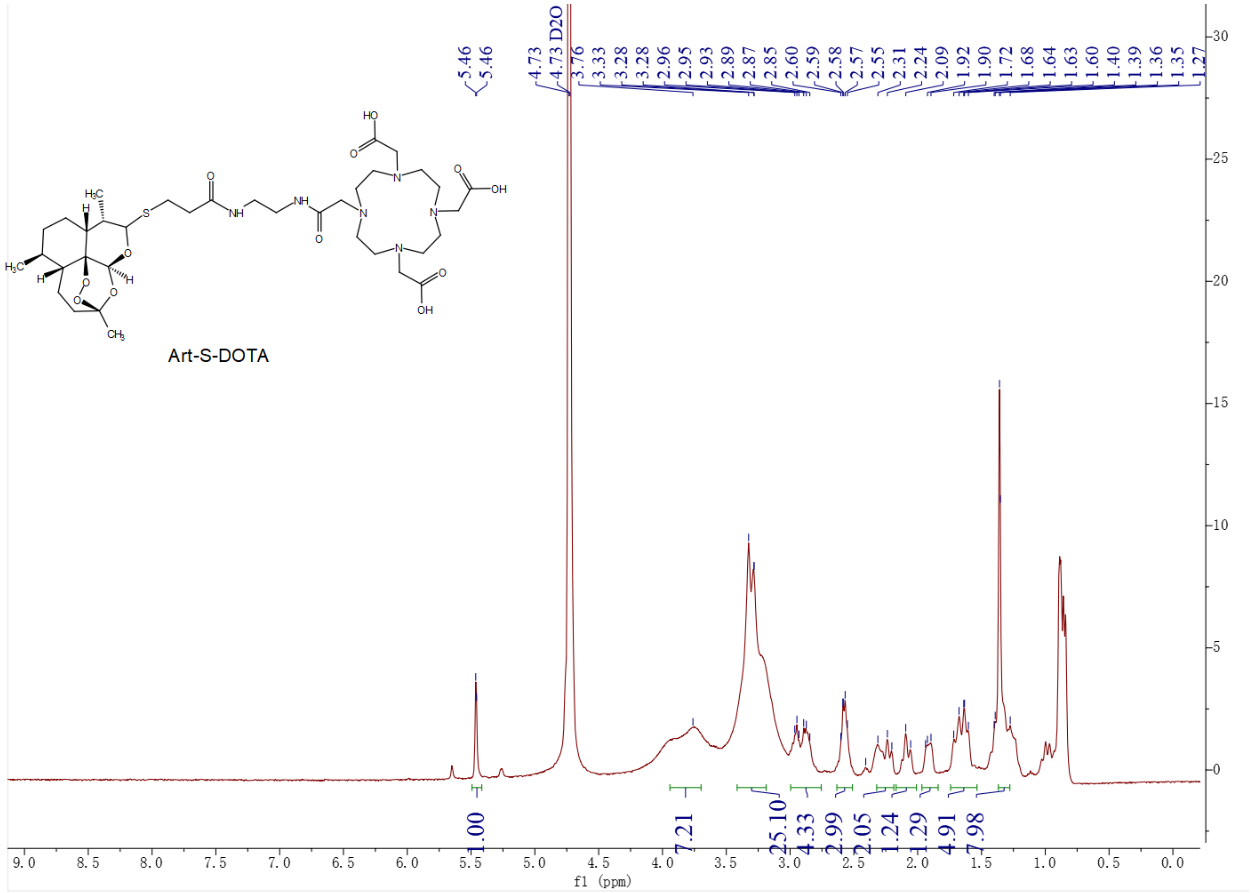

Fig. S4. ^{1}H NMR spectrum of Art-S-DOTA. ^{1}H NMR (400 MHz, D_2O): δ 5.46 (d, J = 2.7 Hz, 1H), 3.76 (s, 7H), 3.41-3.19 (m, 25H), 2.91 (dt, J = 34.1, 8.8 Hz, 4H), 2.58 (q, J = 7.0, 6.6 Hz, 3H), 2.32-2.19 (m, 2H), 2.07 (d, J = 15.4 Hz, 1H), 1.97-1.84 (m, 1H), 1.66 (q, J = 14.4, 12.2 Hz, 5H), 1.36 (d, J = 2.8 Hz, 8H).

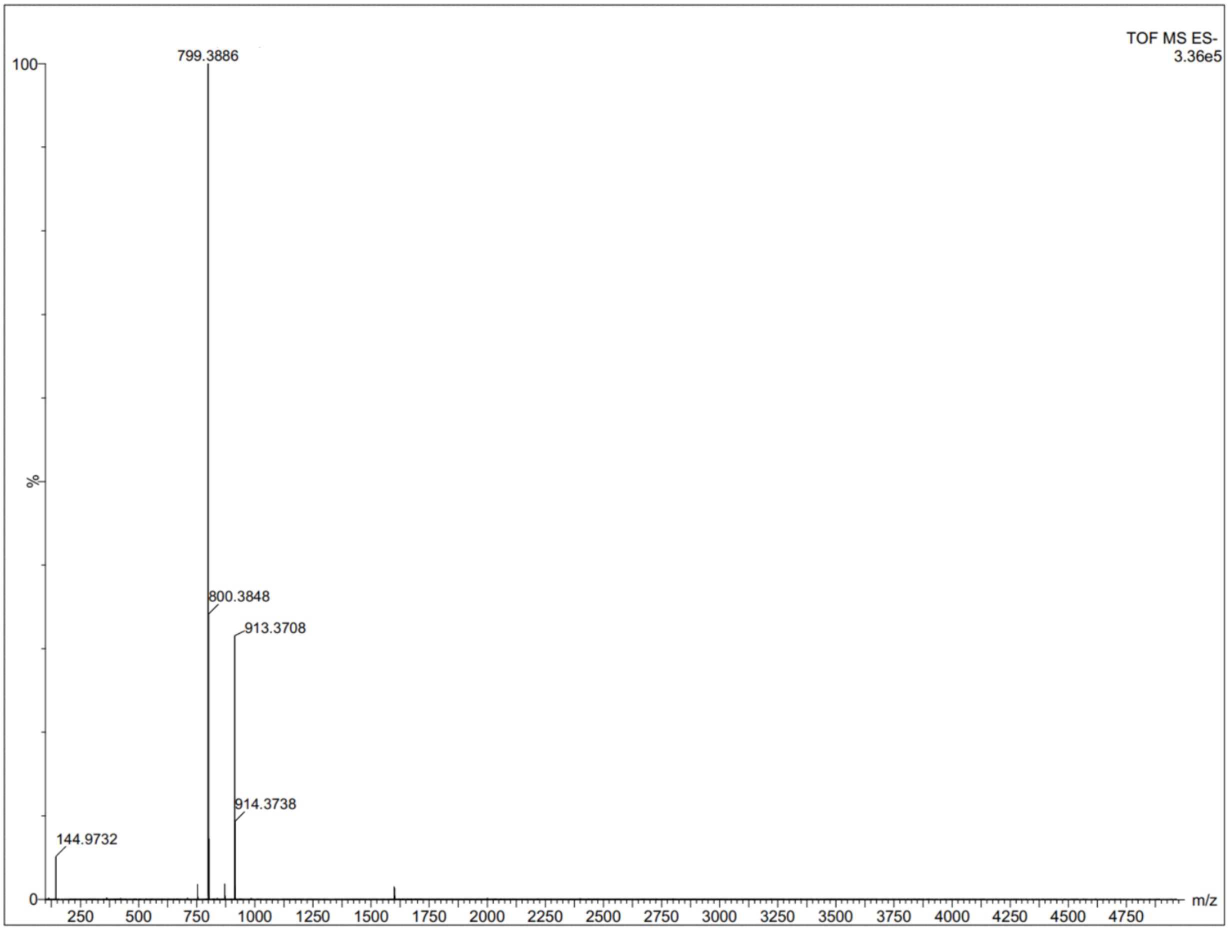

Fig. S5. **MS spectrum of Art-S-DOTA**. ESI-MS (m/z): calcd: 800.3990, found $[M-H]^-$: 799.3886.

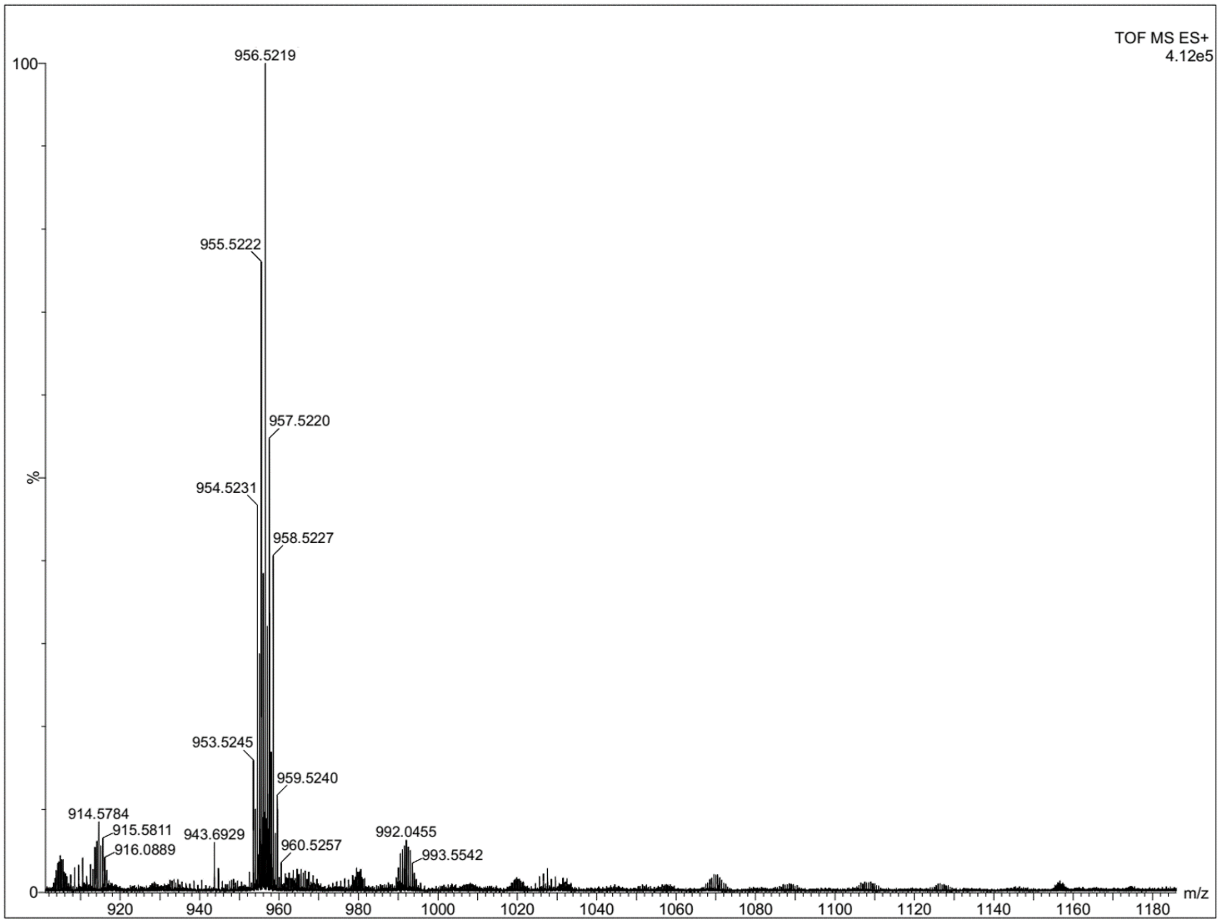

Fig. S6. MS spectrum of Art-Gd. ESI+MS (m/z): calcd: 952.3990~957.3990, found $[M+H]^+$: 953.5249, 954.5231, 955.5222, 956.5219, 957.5220, and 958.5227.

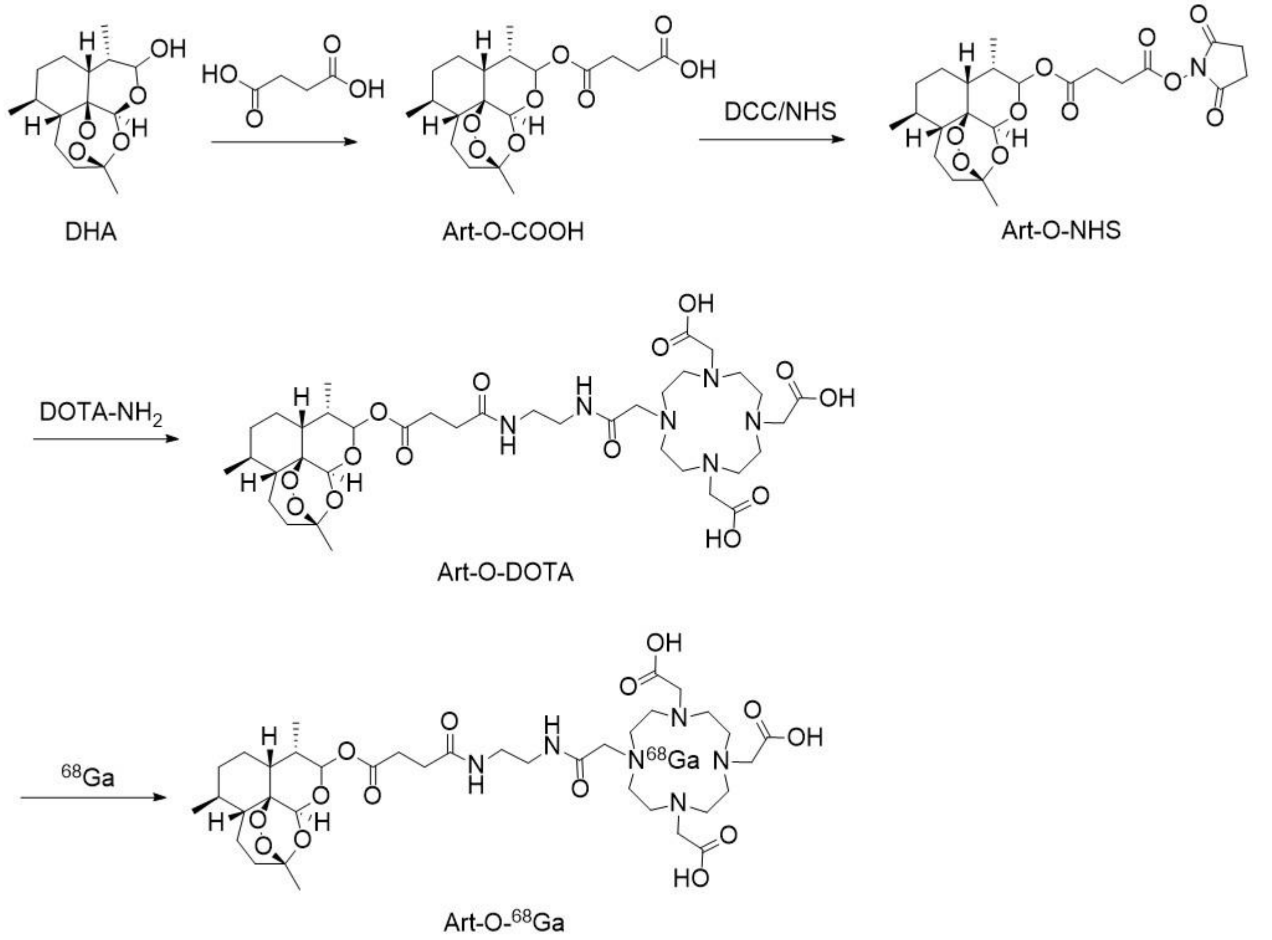

Fig. S7. The synthetic route of the Art-O-^{68}Ga probe. DCC: 1, 3-dicyclohexylcarbodiimide; NHS: N-hydroxysuccinimide, DOTA-NH_2: 2,2’,2’’-(10-(4-((2-aminoethyl)amino)-1-carboxy-4-oxobutyl)-1,4,7,10-tetraazacyclododecane-1,4,7-triyl)triacetic acid.

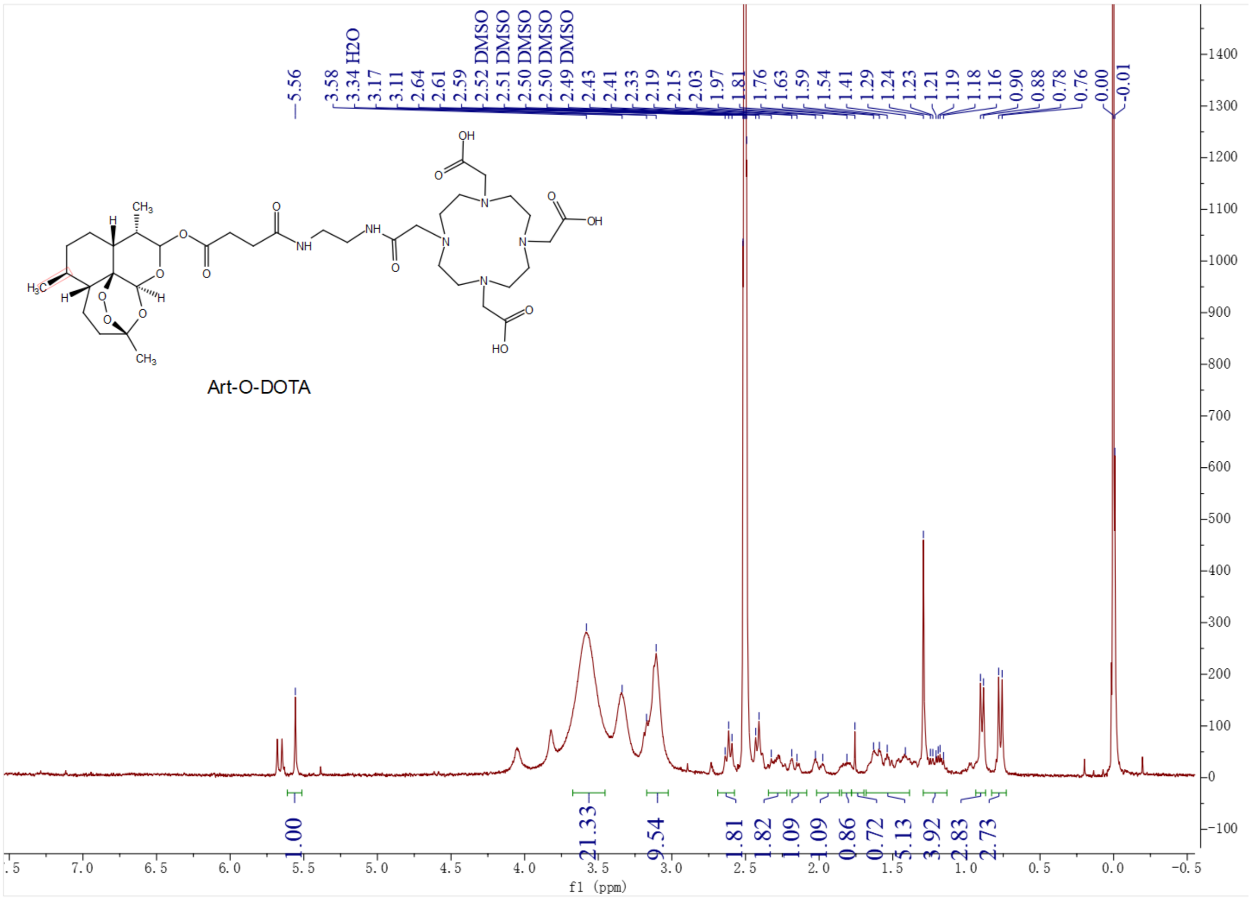

Fig. S8. ^{1}H NMR spectrum of Art-O-DOTA. ^{1}H NMR (400 MHz, DMSO-d_6) δ 5.56 (s, 1H), 3.58 (s, 21H), 3.11 (s, 10H), 2.61 (t, *J* = 7.0 Hz, 2H), 2.17 (d, *J* = 10.6 Hz, 1H), 1.97 (s, 1H), 1.81 (s, 1H), 1.76 (s, 1H), 1.68 – 1.39 (m, 5H), 1.29 (s, 4H), 0.89 (d, *J* = 6.2 Hz, 3H), 0.77 (d, *J* = 7.0 Hz, 3H).

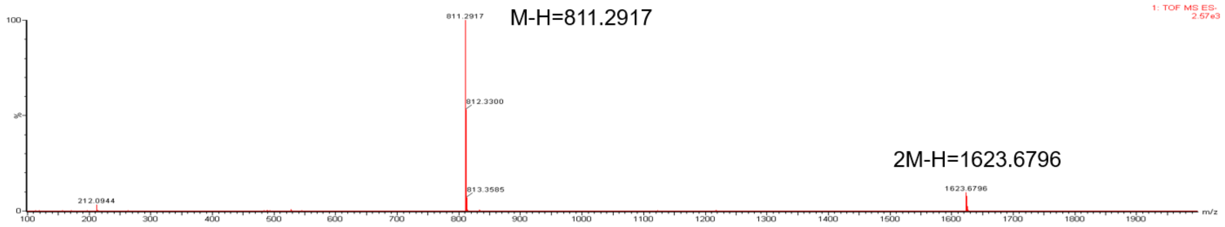

Fig. S9. MS spectrum of Art-O-DOTA. ESI-MS (m/z): calcd: 812.4168, found [M-H]$^-$:811.2917; [2M-H]$^-$: 1623.6786.

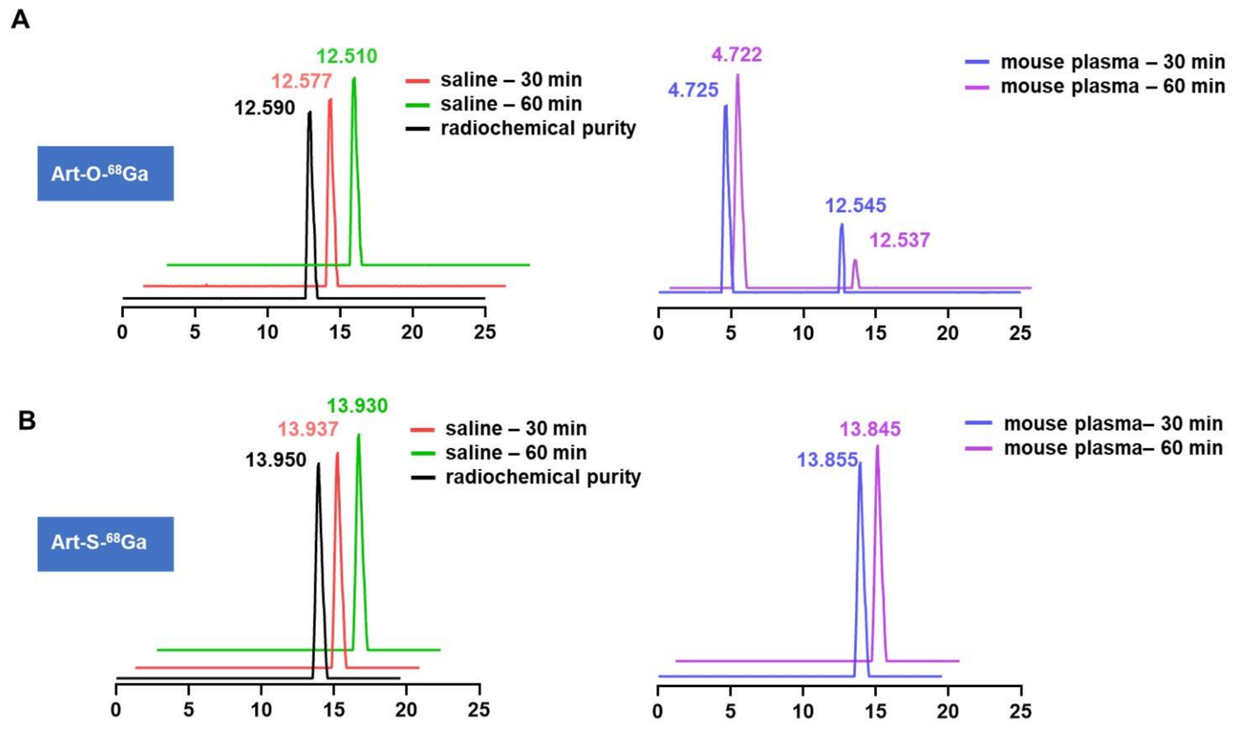

Fig. S10. The stability of the Art-S-^{68}Ga and Art-O-^{68}Ga probe. (A) The stability of the Art-O-^{68}Ga and (B) Art-S-^{68}Ga probe incubated with saline and mouse plasma at 37°C for 30 and 60 min measured by radio-HPLC. The peaks at around 4.7 min represent the DOTA-^{68}Ga, indicating the cleavage of the Art-O-^{68}Ga molecules in the plasma.

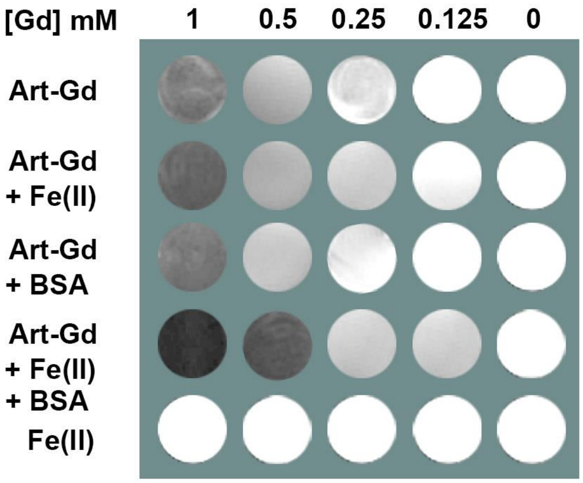

Fig. S11. The *T_2* MRI phantoms the Art-Gd probe. Samples include Art-Gd, Art-Gd + Fe(II), Art-Gd + BSA, Art-Gd + Fe(II) + BSA, and $FeCl_2$ at different concentrations (1, 0.5, 0.25, 0.125, and 0 mM). TR/TE = 2500 / 33 ms.

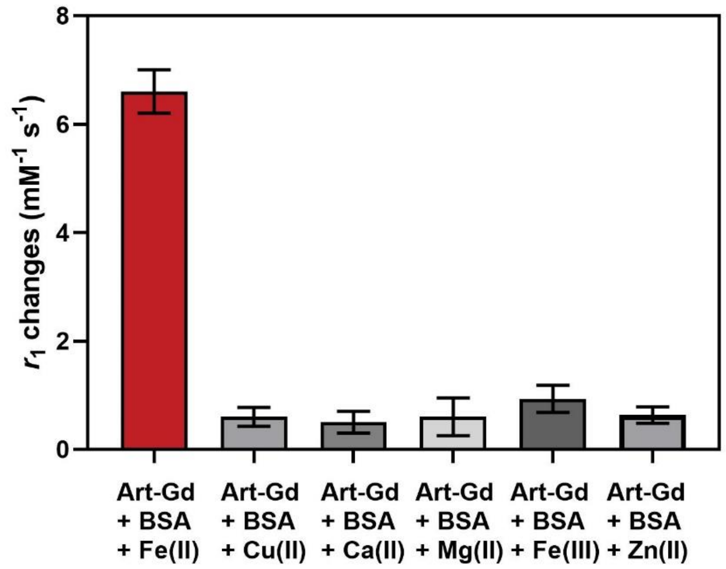

Fig. S12. The selectivity of the Art-Gd probe to major metal ions. Column shows the r_1 relaxivity value changes of the Art-Gd probe treated with the BSA solutions of various metal ions (1 mM), including Fe(II), Cu(II), Ca(II), Mg(II), Fe(III), and Zn(II) ions.

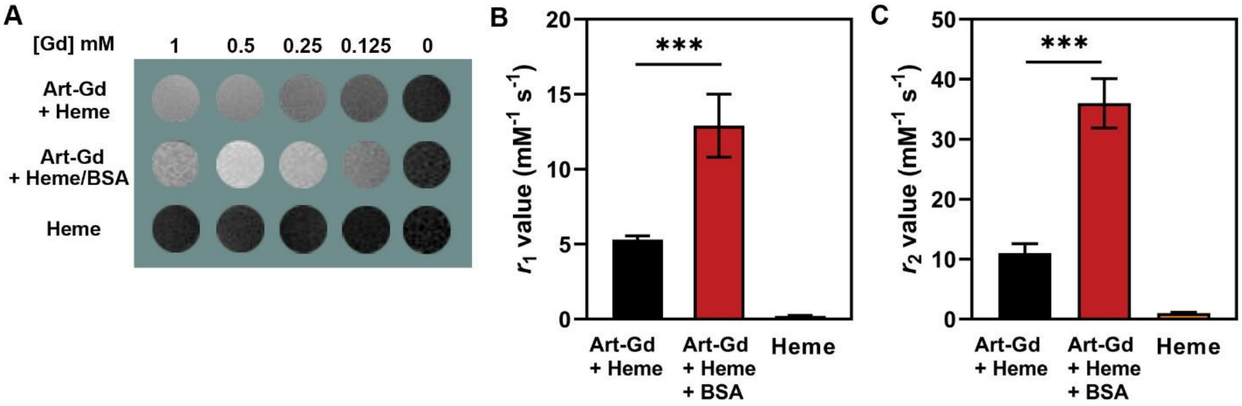

Fig. S13. MRI measurements of the Art-Gd probe incubated with Heme in the presence of BSA. (A) Representative T_1 MRI phantoms of the Art-Gd + Heme, Art-Gd + Heme + BSA, and Heme. (B, C) Column shows the r_1 and r_2 values of the Art-Gd + Heme, Art-Gd + Heme, and Heme. ***$P < 0.001$.

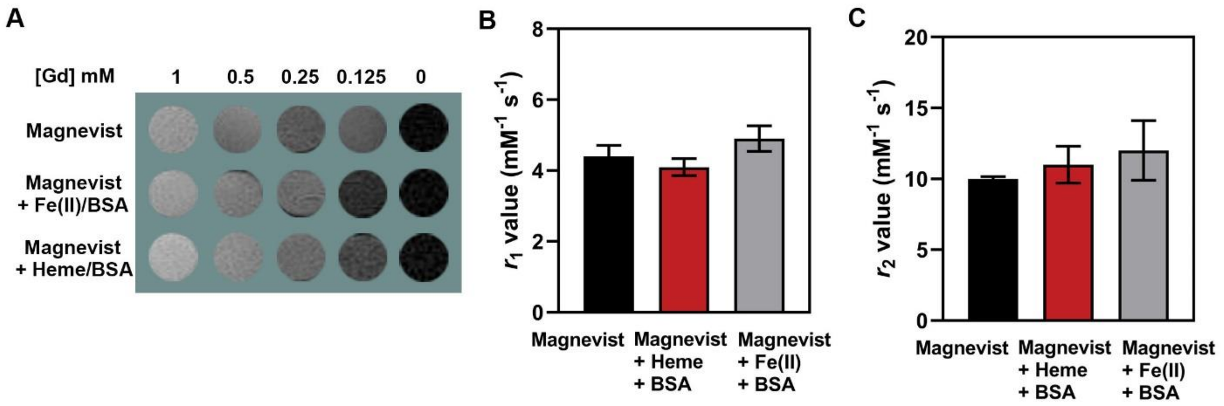

Fig. S14. MRI measurements of Magnevist incubated with Heme/Fe(II) ions in the presence of BSA. (A) Representative T_1 MRI phantoms of Magnevist, Magnevist + Fe(II) + BSA, and Magnevist + Heme + BSA. (B, C) Columns show the r_1 and r_2 values of Magnevist, Magnevist + Fe(II)+ BSA, and Magnevist + Heme + BSA.

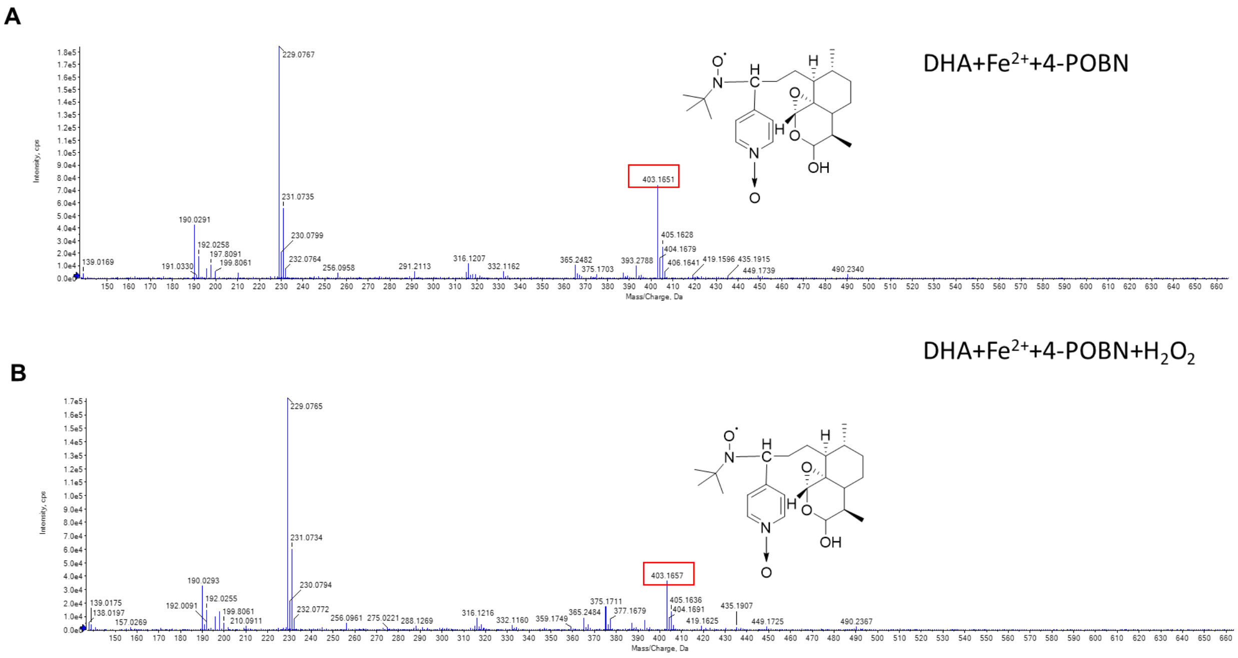

Fig. S15. MS analyses of the mixtures. (A) The mixtures contained Dihydroartemisinin (DHA) (1 mM), $FeCl_2$ (1 mM) and *α*-(4-Pyridyl-1-oxide)-N-tertbutylnitrone (4-POBN) (100 mM). (B) The mixtures contained DHA (1 mM), $FeCl_2$ (1 mM), 4-POBN (100 mM), and H_2O_2 (100 μM). The peak m/z 403 indicates the 4-POBN/DHA radical adducts.

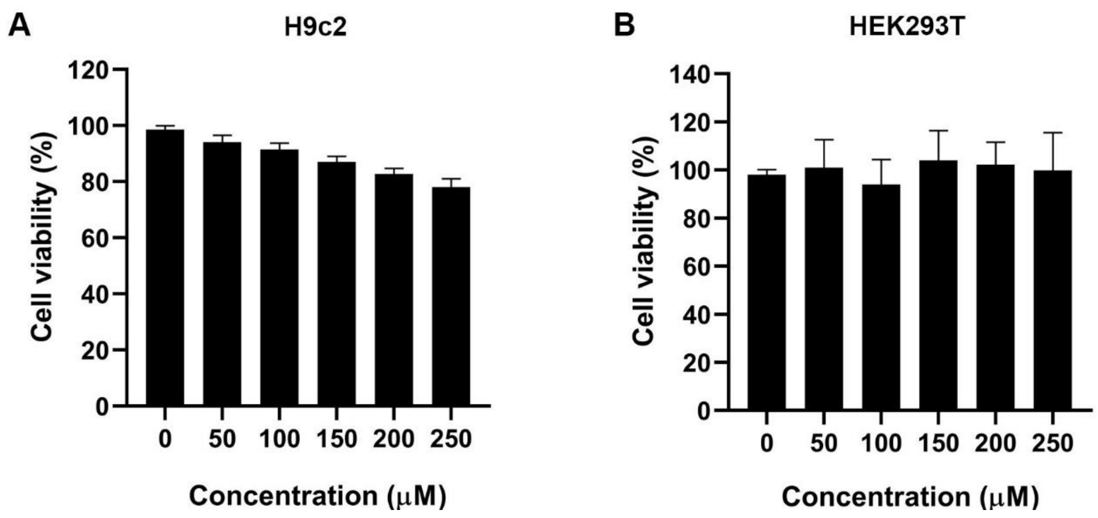

Fig. S16. The cytotoxicity of the Art-Gd molecules. (A, B) The cell viability profiles of H9c2 and HEK293T cells after incubation with different concentrations of the Art-Gd molecules.

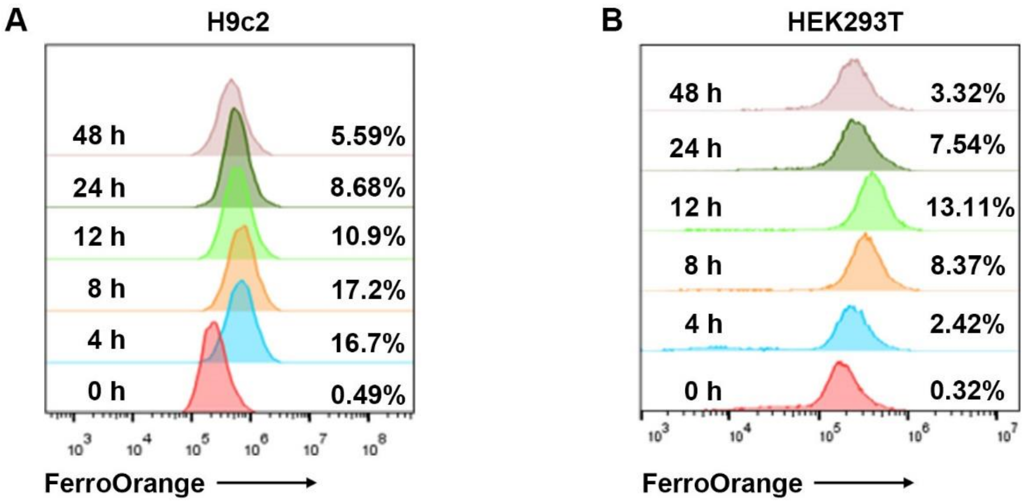

Fig. S17. The level of intracellular Fe(II) ions in H9c2 and HEK293T cells. (A, B) The level of intracellular Fe(II) ions in H9c2 and HEK293T cells after incubation with Erastin (a ferroptosis inducer) was analyzed by flow cytometry using a Fe(II)-sensitive fluorescence probe (FerroOrange).

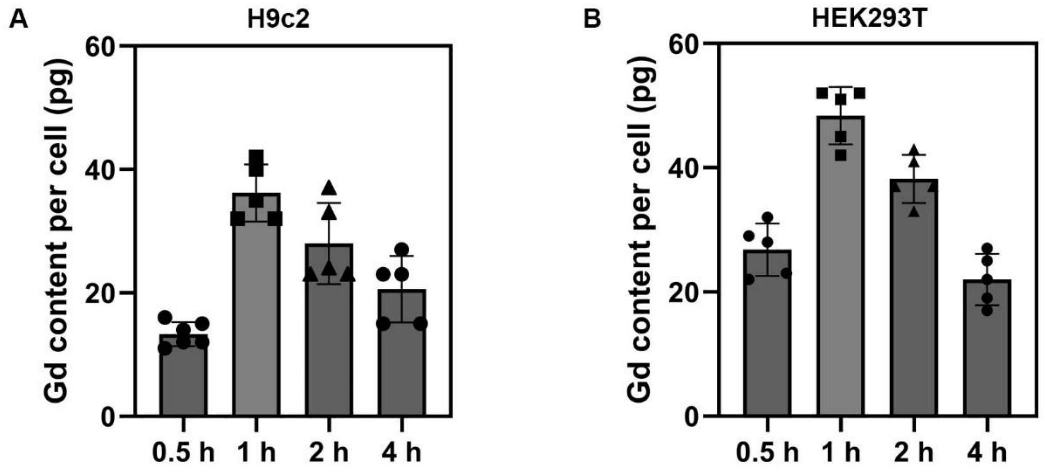

Fig. S18. The administered Gd contents of Erastin-induced H9c2 and HEK293T cells by the Art-Gd probe treatment. (A) Cellular Gd contents of Erastin-induced H9c2 and (B) HEK293T cells after incubation with the Art-Gd probe for different time points (0.5, 1, 2, and 4 h).

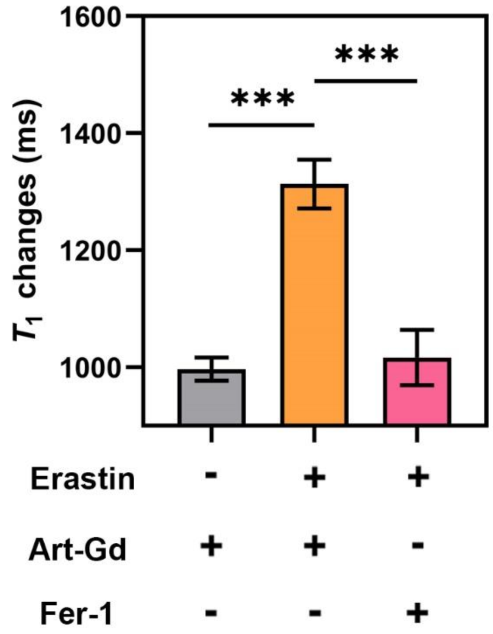

Fig. S19. MRI measurements of the Art-Gd probe in H9c2 cells. Columns show the T_1 relaxation time changes of H9c2 under different treatments. Data represent for mean ± SD. of three independent replicates (n = 3). ***$P < 0.001$.

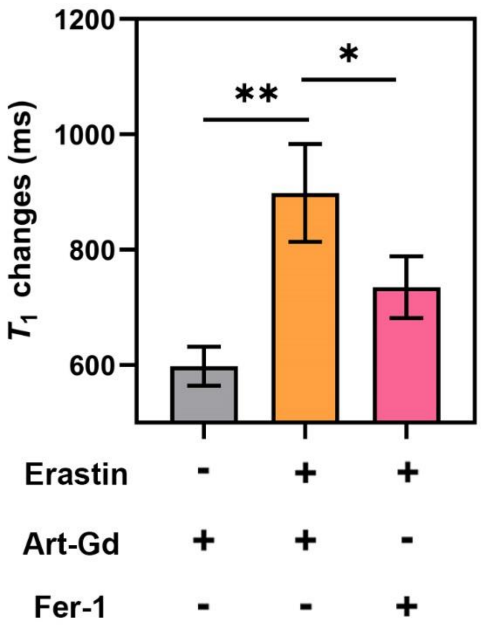

Fig. S20. MRI measurements of the Art-Gd probe in HEK293T cells. Columns show the T_1 relaxation time changes of HEK293T cells under different treatments. Data represent for mean ± SD. of three independent replicates (n = 3). *$P < 0.05$ and **$P < 0.01$.

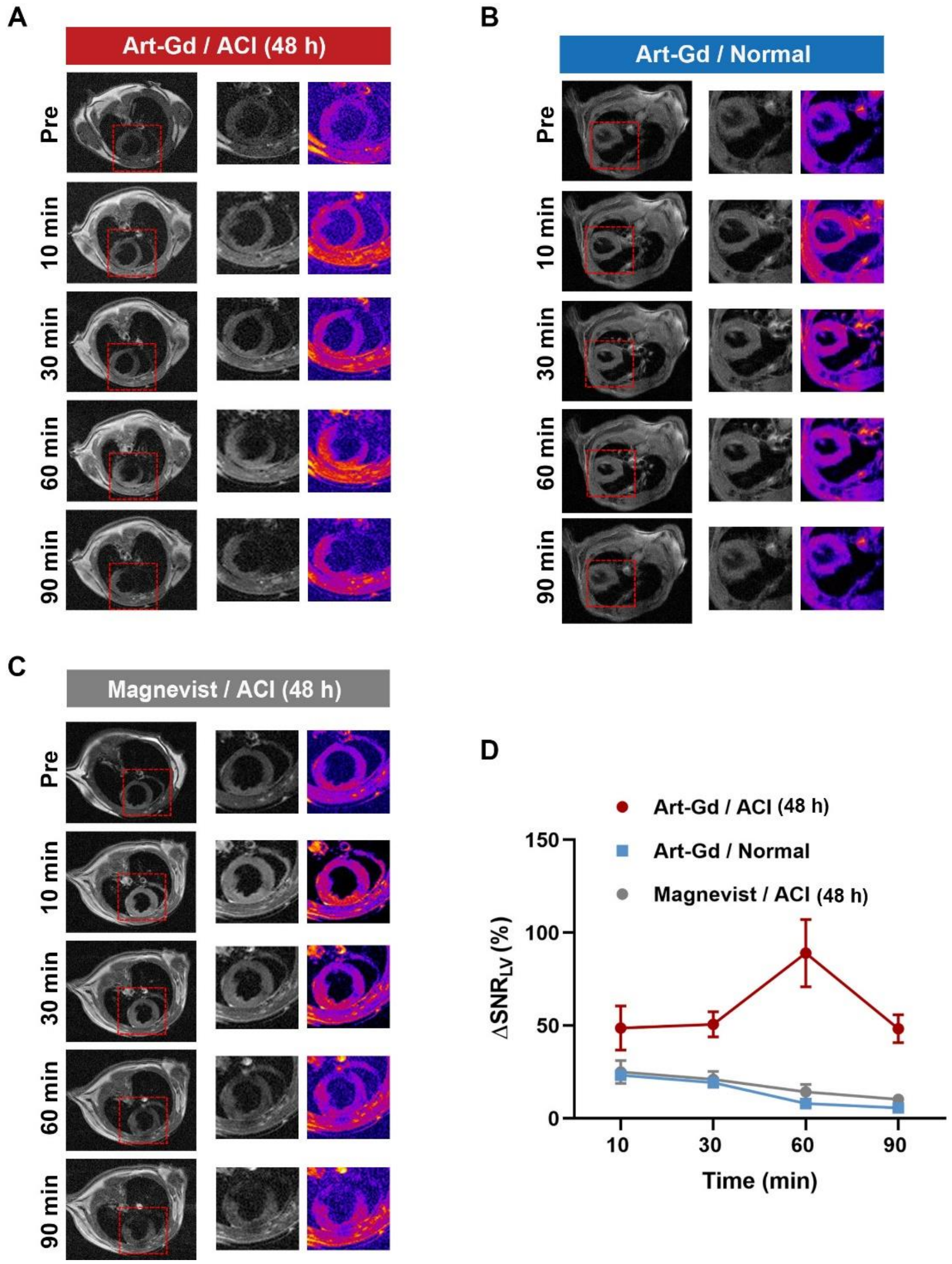

Fig. S21. The feMRI in DOX-induced mouse ACI model (10 mg/kg). (A-C) The T_1-weighted images at 10, 30, 60, and 90 min post-injection of the Art-Gd probe in different groups, including Art-Gd/ACI, Art-Gd/Normal, and Magnevist/ACI, were acquired. Red-dashed circles highlight the region of the axial slice of the heart. (D) The ΔSNR value at 10, 30, 60, and 90 min post-injection of the Art-Gd probe in different groups. $\Delta SNR = (SNR_{pre} - SNR_{post})/SNR_{pre}$.

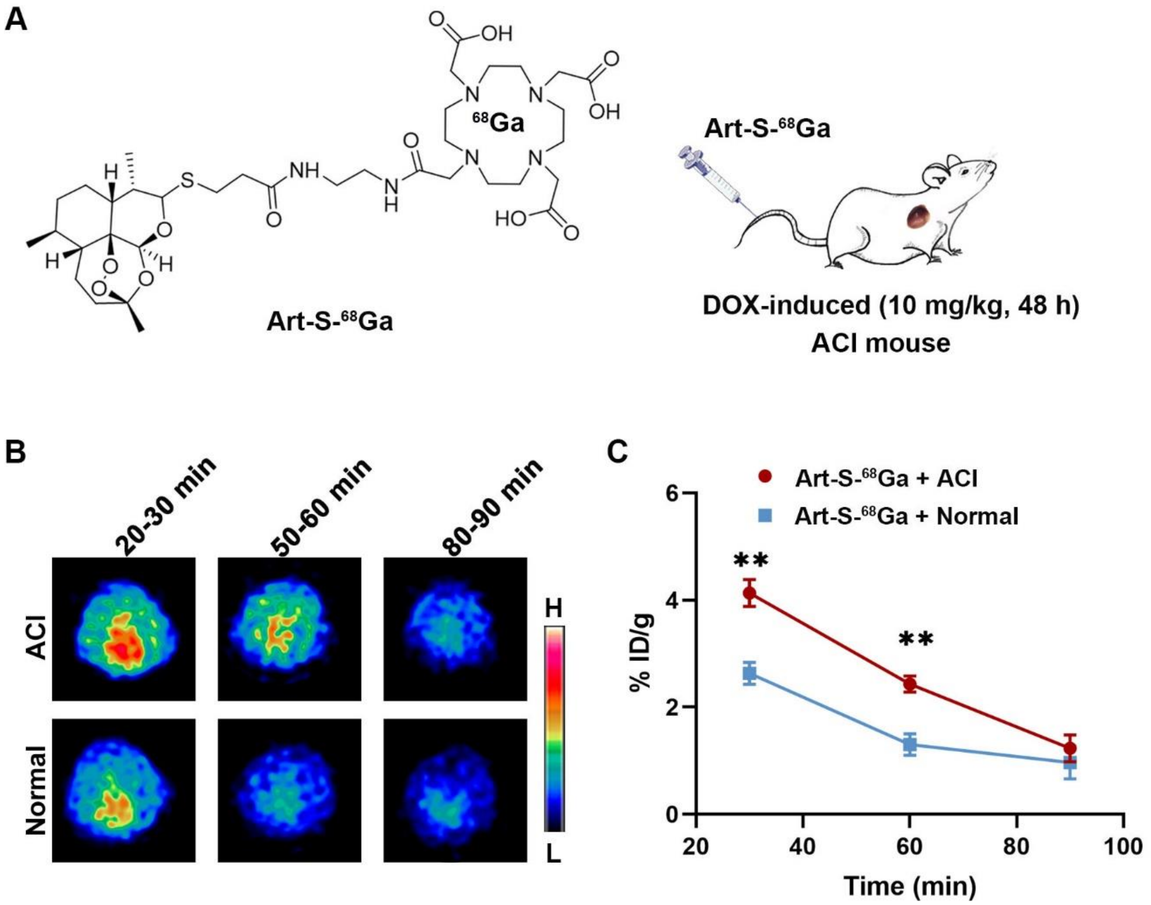

Fig. S22. PET imaging study in DOX-induced mouse ACI model. (A) The chemical structure of the Art-S-^{68}Ga probe and the scheme showing ACI mouse model. (B) PET images of ACI (upper) and normal mice (bottom) (Axial). (C) ROI analysis of the signals in the respective hearts of ACI and normal mice at different time points. $^{**}P < 0.01$.

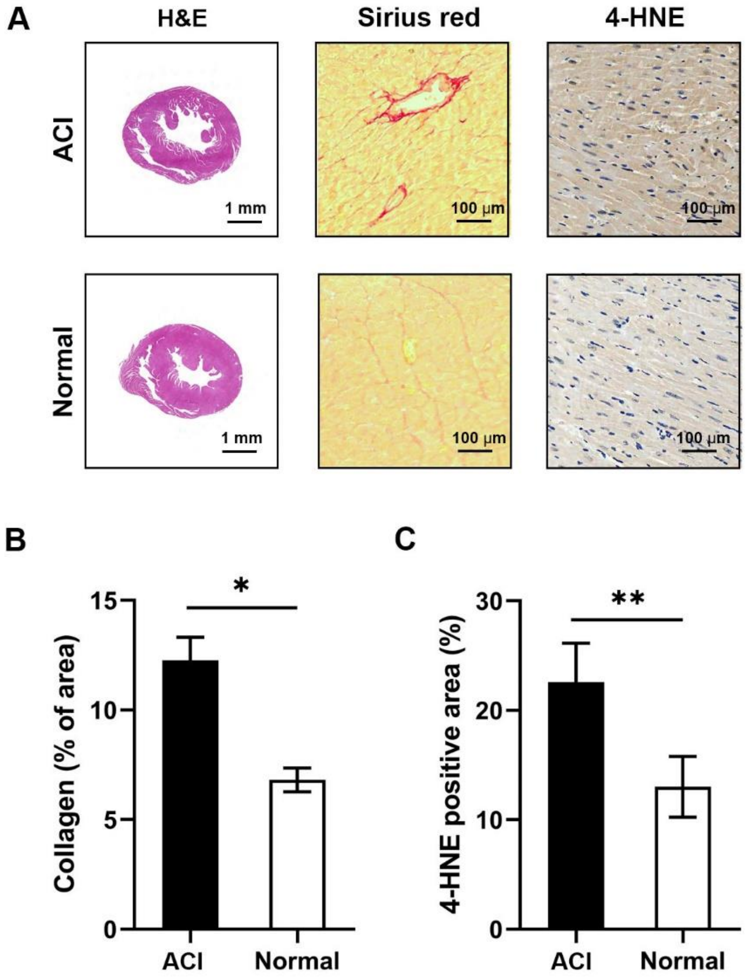

Fig. S23. The histological analysis of DOX-induced ACI mice. (A) Representative images and (B, C) cardiac sections stained with Sirius red (to stain collagen) and 4-HNE positive area (%) in DOX-induced ACI and normal mice (10 mg/kg, i.p., 48 h). *P < 0.05 and **P < 0.01.

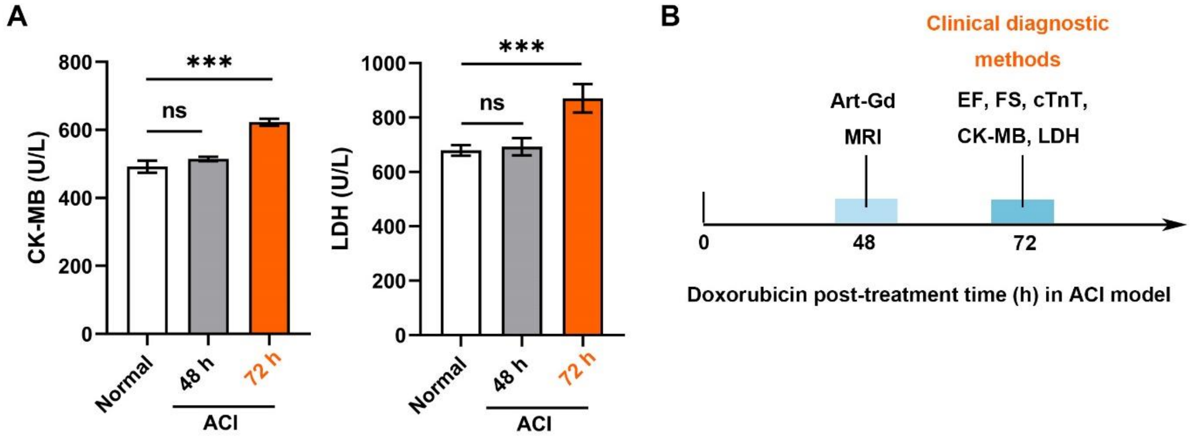

Fig. S24. Evaluation of the cardiac functions at different time points post-treatment with DOX. (A) The changes of LDH and CK-MB in living mice at 0, 48, or 72 h post-treatment of DOX (n = 3). (B) Diagnostic timeline of Art-Gd based MRI detection, clinical diagnostic methods (left ventricular ejection fraction (EF), left ventricular fractional shortening (FS), serum cTnT, CK-MB, and LDH levels) in a DOX-induced ACI mouse model.

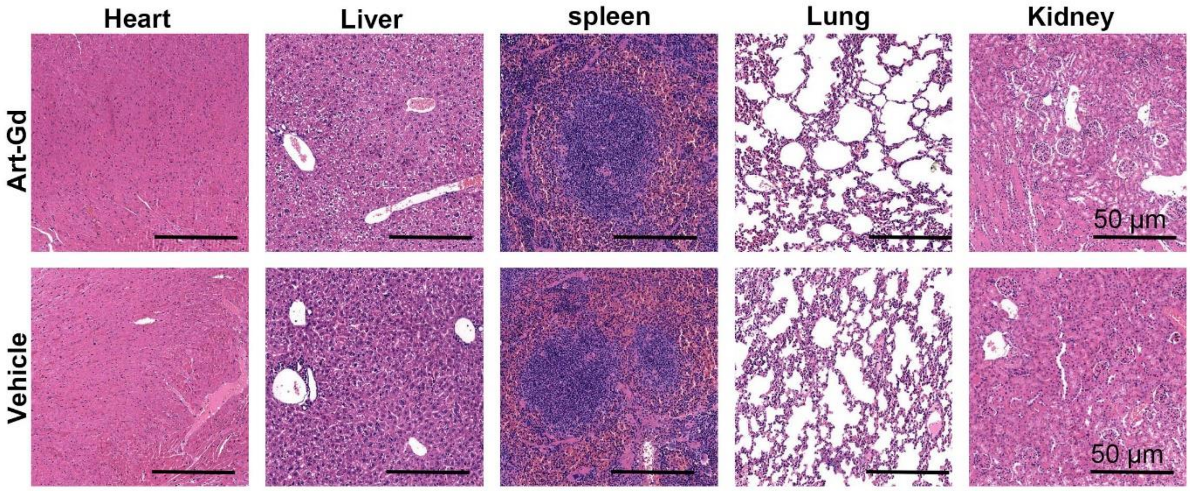

Fig. S25. The H&E staining of major organs after the Art-Gd probe treatments. The major organs were dissected at 24 h after i.v. injection of the Art-Gd probe in normal mice (scale bar = 50 µm).

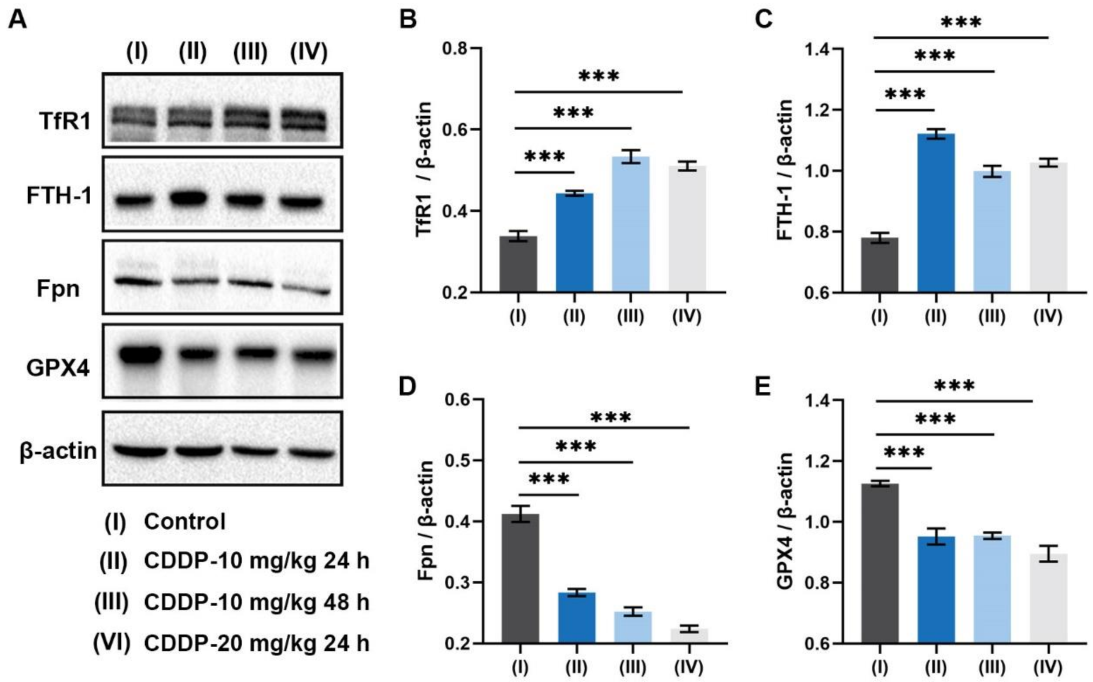

Fig. S26. The Western blotting analysis of ferroptosis-related biomarkers. (A) Representative protein bands and (B) semi-quantitative analysis of TfR1, (C) FTH-1, (D) Fpn, and (E) GPX4 levels in the kidneys from mice treated with CDDP at different doses (10 or 20 mg/kg) and different time points (24 or 48 h). ***P < 0.001.

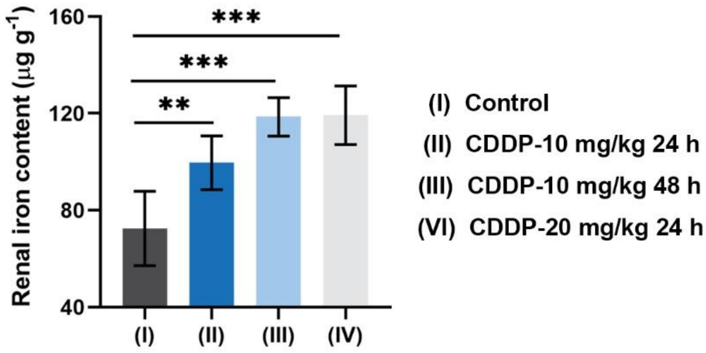

Fig. S27. Total renal Fe content in mice treated with CDDP. Total renal Fe content was measured 24 or 48 h after CDDP (10 or 20 mg/kg) or saline treatment (n = 3). **P < 0.01 and ***P < 0.001.

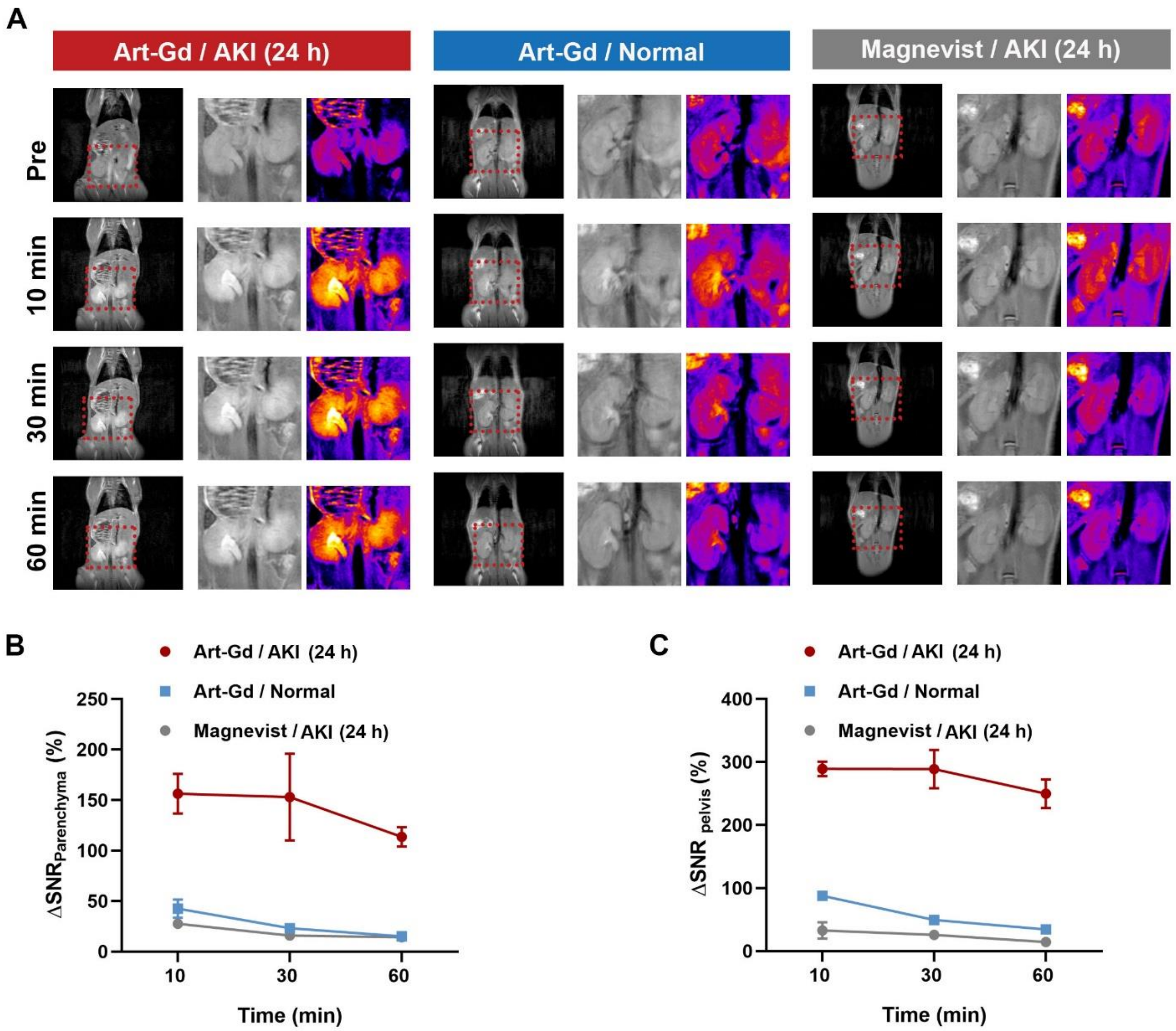

Fig. S28. The feMRI in CDDP-induced AKI mouse model (20 mg/kg). (A) The representative T_1-weighted images at 10, 30, and 60min post-injection of the Art-Gd probe in different groups, including Art-Gd/AKI, Art-Gd/Normal, and Magnevist/AKI, were acquired. Red dashed circles highlight the region of the coronal slice of the kidney. (B, C) The ΔSNR value of parenchyma or pelvis at 10, 30, 60, and 90 min post-injection of the Art-Gd probe in different groups. $\Delta SNR = (SNR_{pre} - SNR_{post})/SNR_{pre}$.

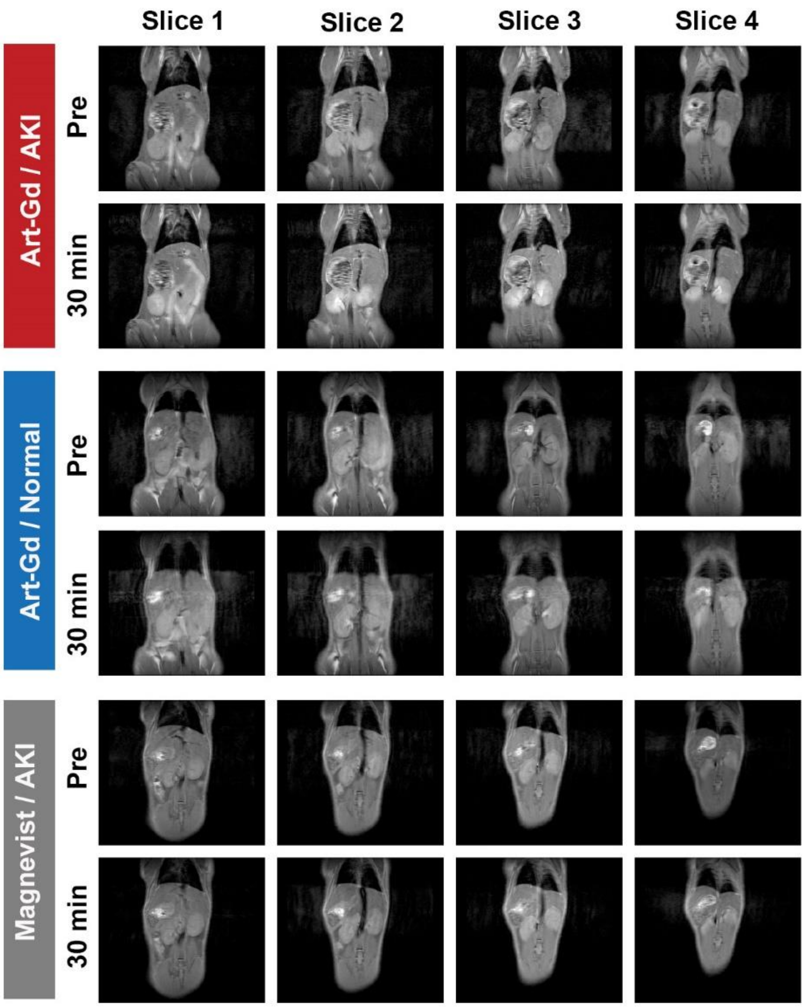

Fig. S29. Additional multi-slice T_1-weighted images of CDDP-induced AKI mouse model (20 mg/kg). The multi-slice representative T_1-weighted images at 30 min post-injection of the Art-Gd probe were acquired.

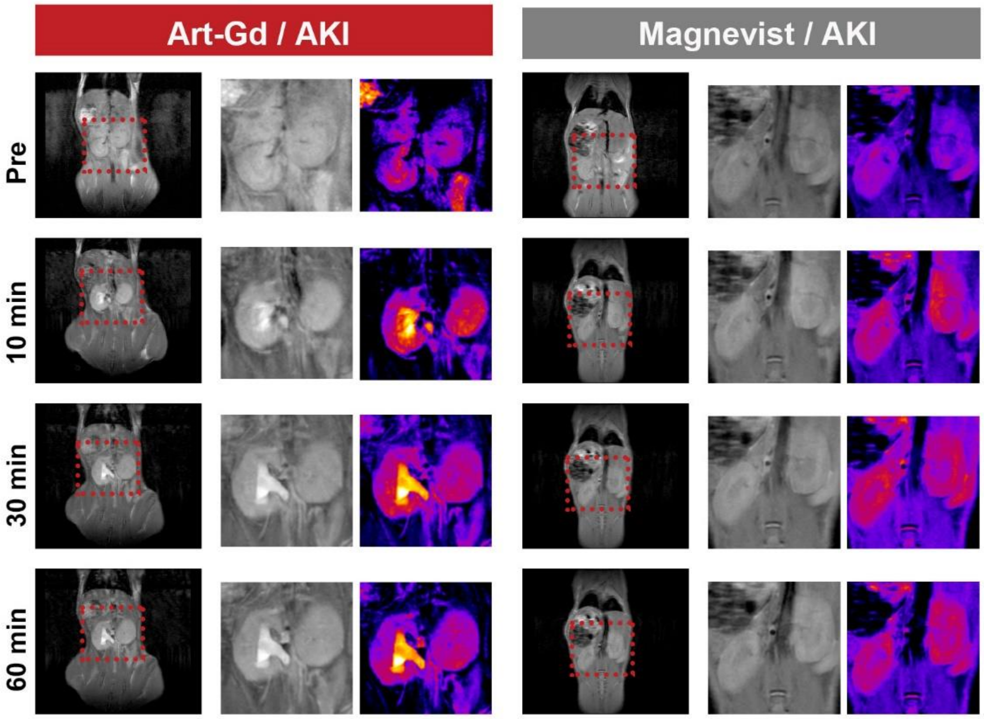

Fig. S30. The feMRI in CDDP-induced AKI mouse model (10 mg/kg). The T_1 MRI study of CDDP-induced AKI mice (10 mg/kg, 24 h). The representative T_1-weighted images at 10, 30, and 60 min post-injection of the Art-Gd probe were acquired. Red dashed circles highlight the region of the coronal slice of the kidney.

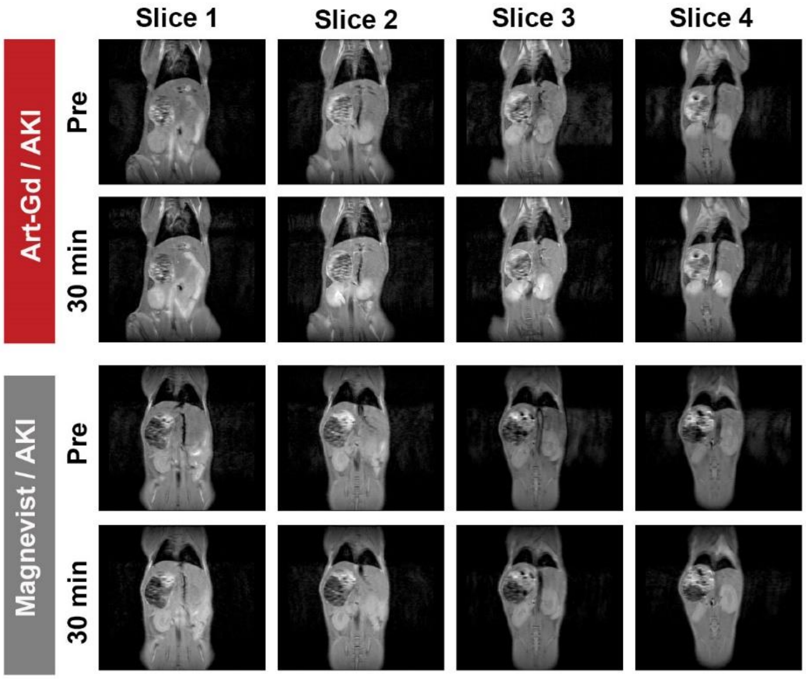

Fig. S31. Additional multi-slice T_1-weighted images of CDDP-induced AKI mouse model (10 mg/kg). The multi-slice representative T_1-weighted images at 30 min post-injection of the Art-Gd probe were acquired.

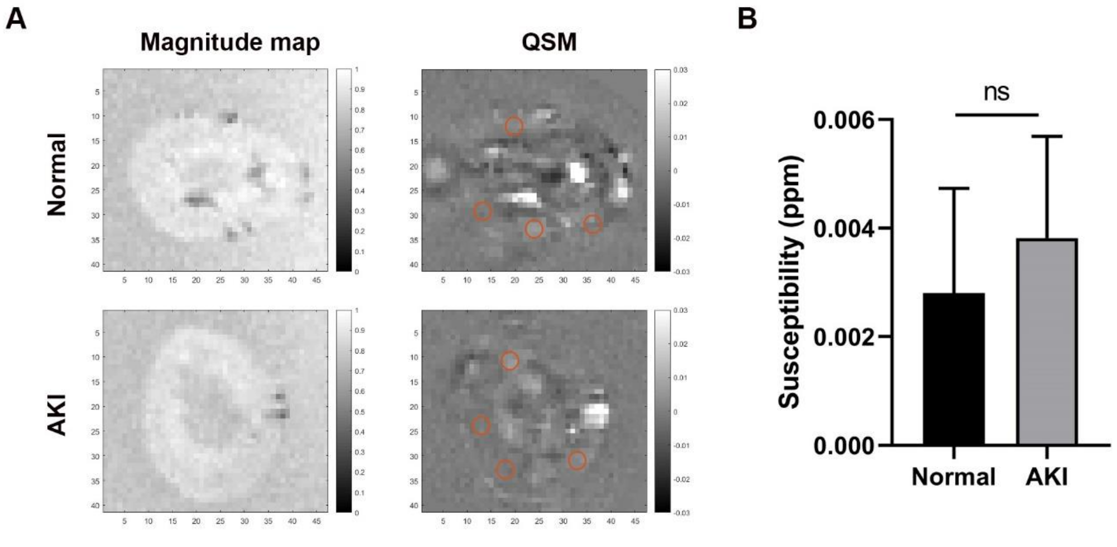

Fig. S32. Magnetic susceptibility imaging. (A) Magnitude maps and quantitative susceptibility maps (QSM) of the kidneys from CDDP-induced AKI (20 mg/kg, 24 h) and saline-treated mice under a single scan. (B) Quantitative analysis of the magnetic susceptibility of the CDDP-treated kidney and saline-treated kidney. Susceptibility values were measured from regions of interest (orange circles).

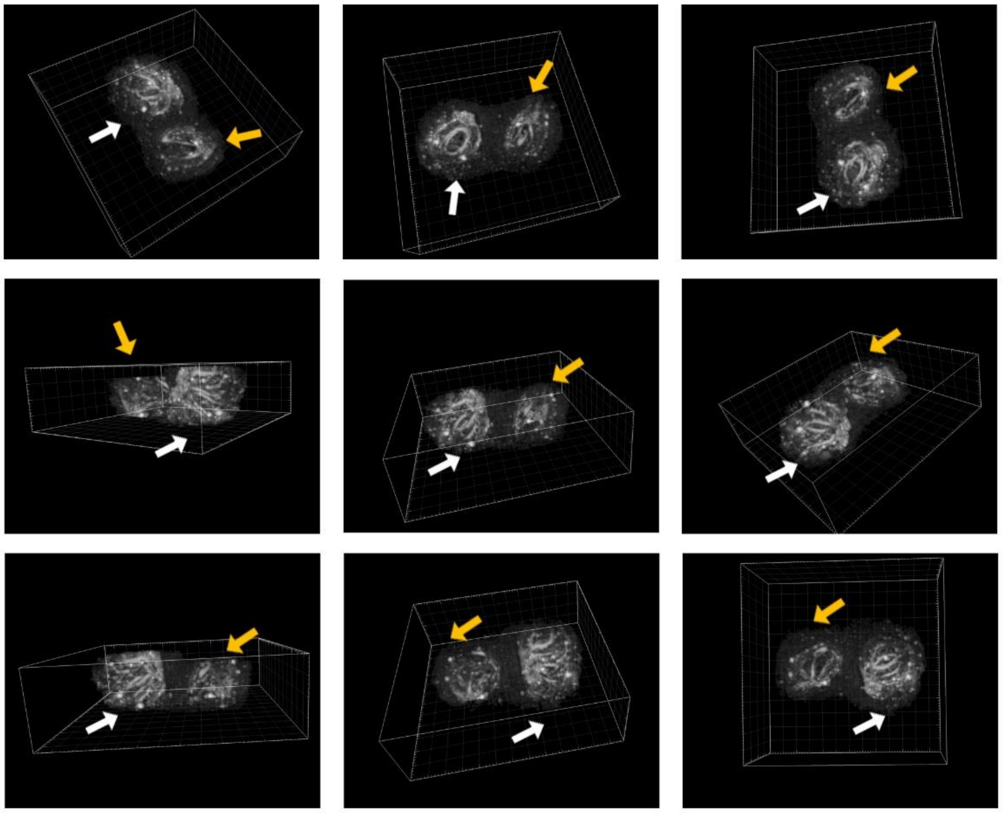

Fig. S33. Representative figures from the reconstructed dynamic 3D. White arrows indicate CDDP-treated kidney tissue and yellow arrows indicate normal kidney tissue.

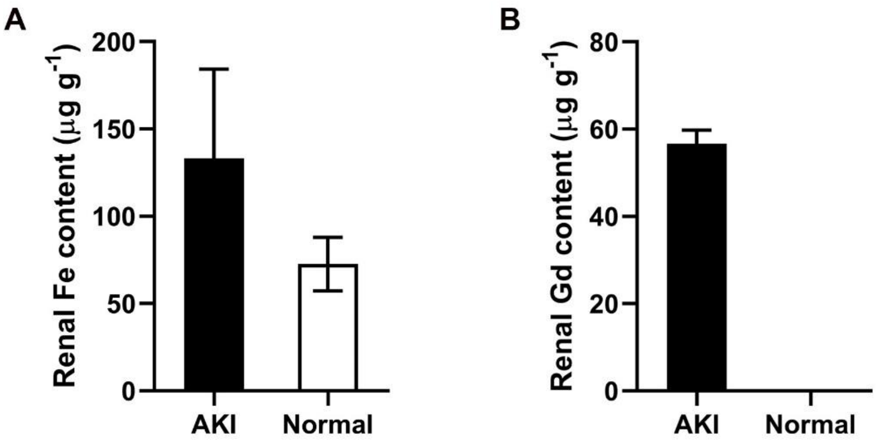

Fig. S34. Total renal Fe and Gd contents at 24 h post-treatment with CDDP (20 mg/kg, 24 h). (A) Total renal Fe and (B) Gd contents in CDDP-induced AKI mice (20 mg/kg, 24 h) and saline-treated mice (*n* = 3).

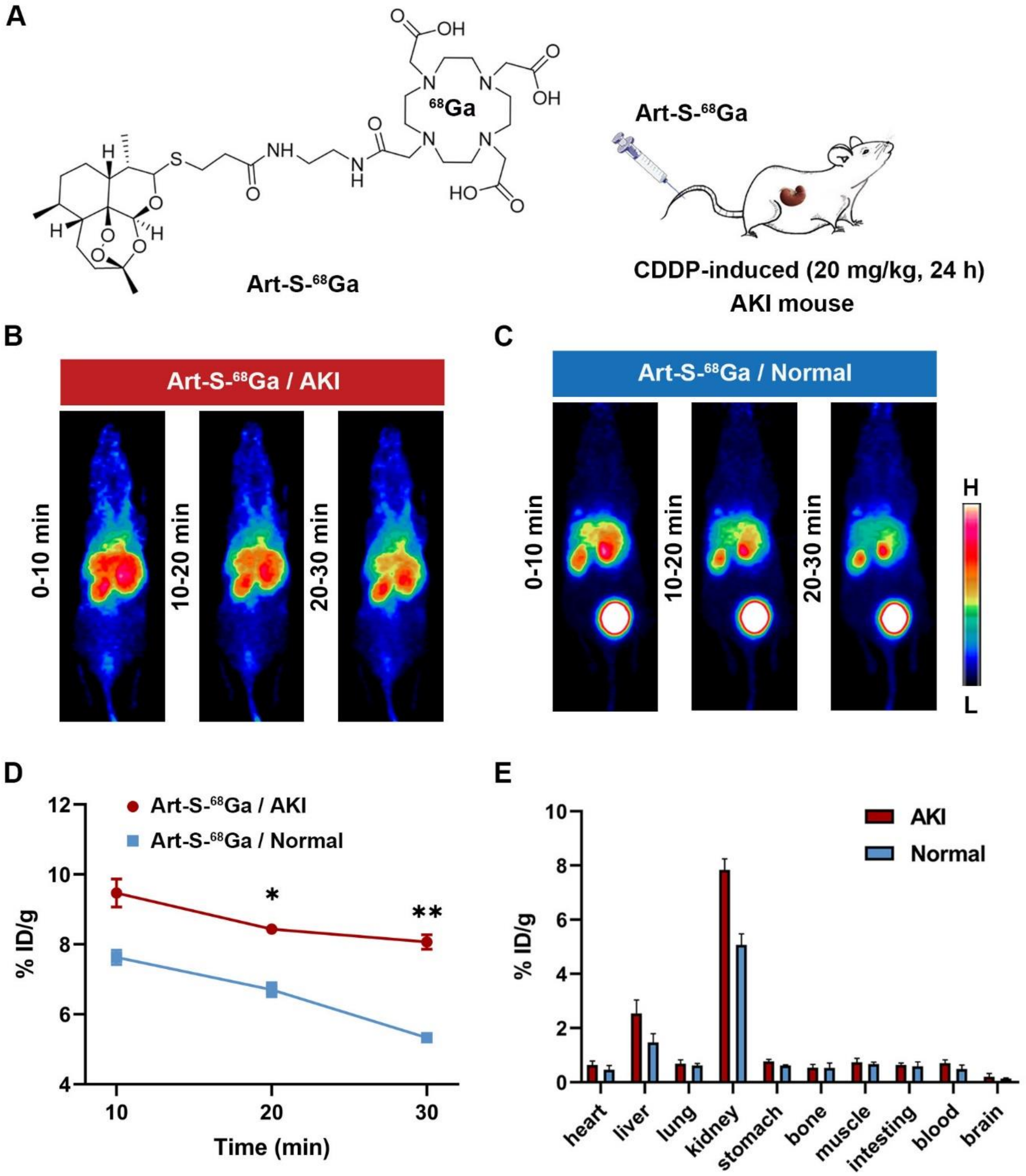

Fig. S35. PET imaging study in CDDP-induced AKI mouse model (20 mg/kg). (A) The chemical structure of the Art-S-^{68}Ga probe and the scheme showing AKI mouse model. (B, C) The PET images of AKI (left) and normal mice (right). (D) ROI analysis of the signals in the respective kidneys of AKI and normal mice at different time points (n = 3). (E) Biodistribution of Art-S-^{68}Ga in AKI and normal mice at 30 min post-injection. *$P < 0.05$ and **$P < 0.01$.

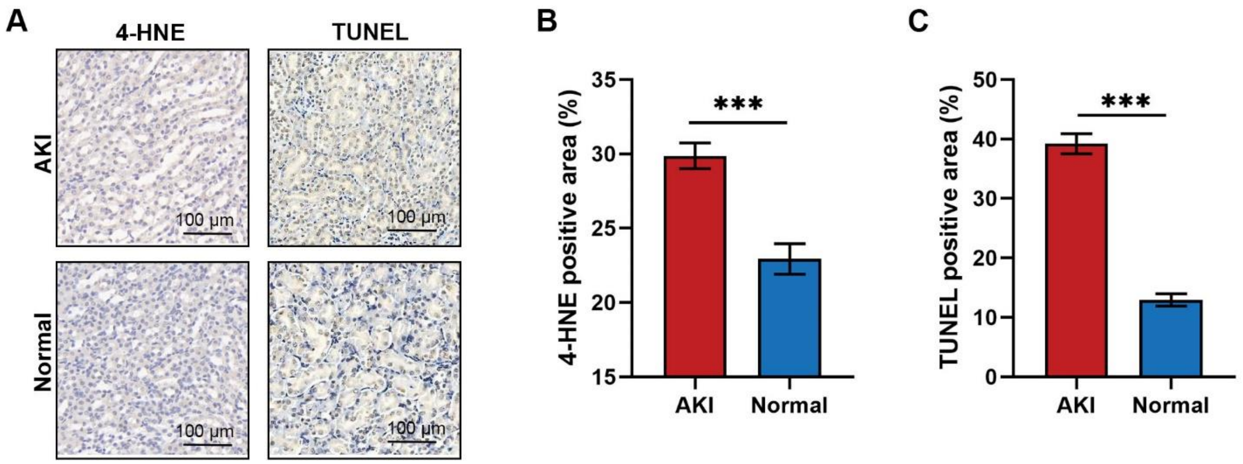

Fig. S36. The histological analysis of CDDP-induced AKI mice. (A) Representative images (scale bar = 100 μm) and (B, C) quantitative analyses of 4-HNE and TUNEL positive area (%) in kidney sections of CDDP-induced AKI (20 mg/kg, 24 h) and normal mice. ****P* < 0.001.

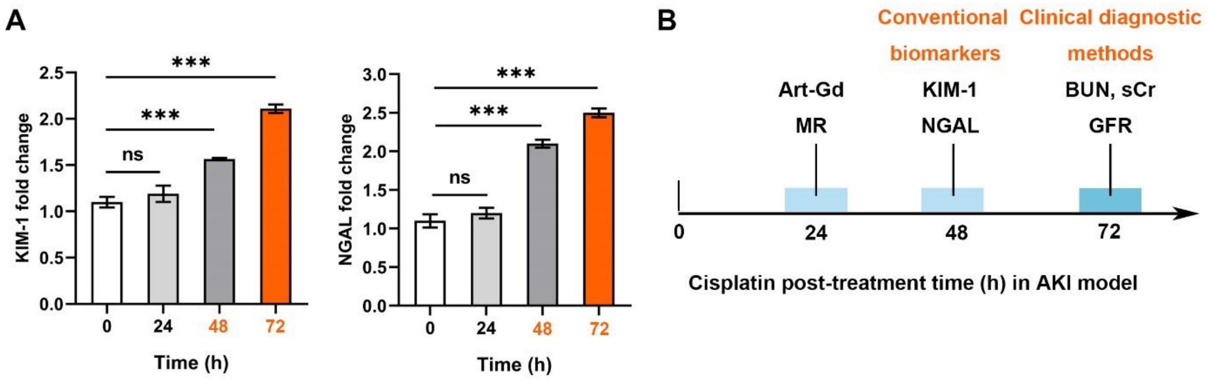

Fig. S37. Evaluation of the kidney functions at different time points post-treatment with CDDP. (A) The changes of KIM-1 and NGAL in living mice at 0, 24, 48, or 72 h post-treatment of CDDP (*n* = 3). (B) Diagnostic timeline of Art-Gd-based MRI detection, conventional biomarkers (NGAL and KIM-1), and clinical diagnostic methods (sCr, BUN, and GFR) in a CDDP-induced AKI mouse model.

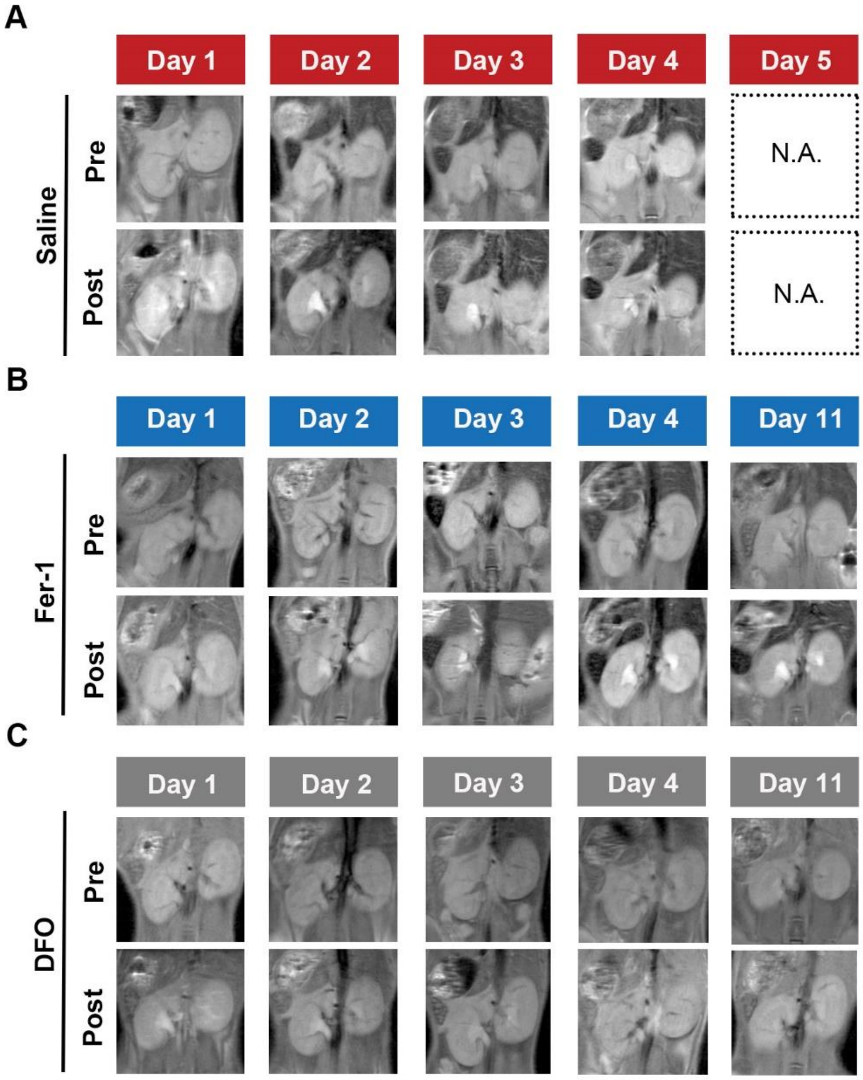

Fig. S38. The MR images of evaluation the therapeutic efficacy in CDDP-induced AKI mouse models. (A-C) Representative T_1-weighted images of mouse kidneys from different treatments (saline, Fer-1, and DFO) at pre- and post-contrast points were acquired on day 1, 2, 3, 4, or 11 after treatment with CDDP. N.A. represents to not available due to the death of the mouse group.

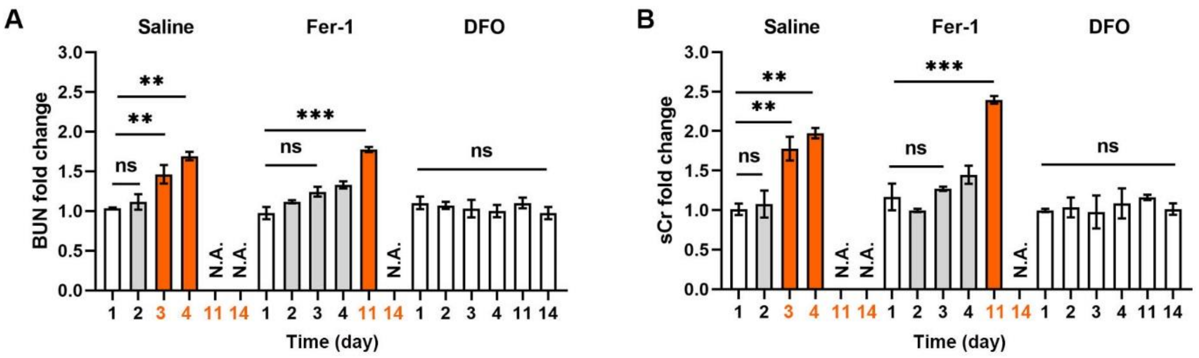

Fig. S39. Detection of kidney function with BUN and sCr assays. (A, B) The fold-changes of the BUN and sCr in living mice (*n* = 5) after different treatments at different time points of days 1, 2, 3, 4, 11, and 14. The ns represents for no significance. ***P* < 0.01 and ****P* < 0.001.

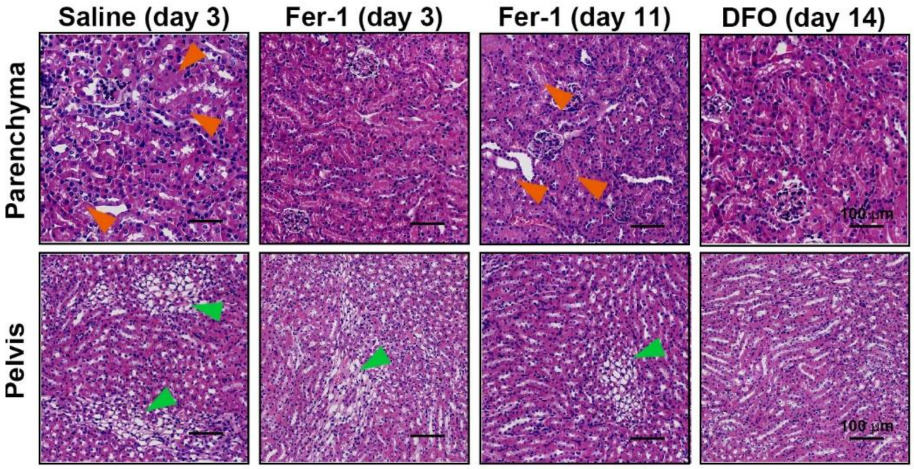

Fig. S40. Representative H&E staining results of the pelvis and parenchyma region of kidneys with different treatments. The mouse groups were pre-treated with saline, Fer-1, or DFO before CDDP treatment (20 mg/kg). Orange arrows indicate damaged tubules and green arrows indicate the infiltration of multifocal foam cells. Scale bar = 100 µm for all images.

	r_1 (mM^{-1} s^{-1})	r_2 (mM^{-1} s^{-1})	r_2/r_1
**Art-Gd**	5.10 ± 0.30	11.90 ± 1.31	2.34 ± 0.34
**Art-Gd + Fe(II)**	5.20 ± 0.40	14.00 ± 1.47	2.69 ± 0.44
**Art-Gd + BSA**	6.30 ± 0.60	13.56 ± 2.22	2.22 ± 0.17
**Art-Gd + Fe(II) +BSA**	10.70 ± 0.60	33.20 ± 4.60	2.98 ± 0.49

Table S1. The r_2/r_1 values of the Art-Gd probe in various formulas. These values were measured at a 9.4 T MRI scanner based on independent triplicate experiments.
